# Gene4PD: A Comprehensive Genetic Database of Parkinson’s Disease

**DOI:** 10.3389/fnins.2021.679568

**Published:** 2021-04-26

**Authors:** Bin Li, Guihu Zhao, Qiao Zhou, Yali Xie, Zheng Wang, Zhenghuan Fang, Bin Lu, Lixia Qin, Yuwen Zhao, Rui Zhang, Li Jiang, Hongxu Pan, Yan He, Xiaomeng Wang, Tengfei Luo, Yi Zhang, Yijing Wang, Qian Chen, Zhenhua Liu, Jifeng Guo, Beisha Tang, Jinchen Li

**Affiliations:** ^1^National Clinical Research Center for Geriatric Disorders, Department of Geriatrics, Xiangya Hospital, Central South University, Changsha, China; ^2^Department of Neurology, Xiangya Hospital, Central South University, Changsha, China; ^3^Mobile Health Ministry of Education—China Mobile Joint Laboratory, Xiangya Hospital, Central South University, Changsha, China; ^4^Center for Medical Genetics, Hunan Key Laboratory, School of Life Sciences, Central South University, Changsha, China; ^5^Department of Pathogen Biology, School of Basic Medical Sciences, Central South University, Changsha, China

**Keywords:** Parkinson’s disease, PD-associated genes, Gene4PD database, age at onset, genetic variant

## Abstract

Parkinson’s disease (PD) is a complex neurodegenerative disorder with a strong genetic component. A growing number of variants and genes have been reported to be associated with PD; however, there is no database that integrate different type of genetic data, and support analyzing of PD-associated genes (PAGs). By systematic review and curation of multiple lines of public studies, we integrate multiple layers of genetic data (rare variants and copy-number variants identified from patients with PD, associated variants identified from genome-wide association studies, differentially expressed genes, and differential DNA methylation genes) and age at onset in PD. We integrated five layers of genetic data (8302 terms) with different levels of evidences from more than 3,000 studies and prioritized 124 PAGs with strong or suggestive evidences. These PAGs were identified to be significantly interacted with each other and formed an interconnected functional network enriched in several functional pathways involved in PD, suggesting these genes may contribute to the pathogenesis of PD. Furthermore, we identified 10 genes were associated with a juvenile-onset (age ≤ 30 years), 11 genes were associated with an early-onset (age of 30–50 years), whereas another 10 genes were associated with a late-onset (age > 50 years). Notably, the AAOs of patients with loss of function variants in five genes were significantly lower than that of patients with deleterious missense variants, while patients with *VPS13C* (*P* = 0.01) was opposite. Finally, we developed an online database named Gene4PD (http://genemed.tech/gene4pd) which integrated published genetic data in PD, the PAGs, and 63 popular genomic data sources, as well as an online pipeline for prioritize risk variants in PD. In conclusion, Gene4PD provides researchers and clinicians comprehensive genetic knowledge and analytic platform for PD, and would also improve the understanding of pathogenesis in PD.

## Introduction

Parkinson’s disease (PD) is the second most common neurodegenerative disease (Alzheimer’s Association, 2014; Dorsey et al., 2007). PD, particularly early-onset disease, shows high heritability (Blauwendraat et al., 2019a), suggesting that genetic factors play important roles in its pathogenesis. In the last two decades with the development of high throughput technologies including next-generation sequencing (Jansen et al., 2017; Sandor et al., 2017; Siitonen et al., 2017), numerous studies have focused on the genetic architectures to understand the pathogenesis of PD (Blauwendraat et al., 2019b; Kalia and Lang, 2015; Labbe et al., 2016). Several rare variants and candidate genes have been identified among familial and sporadic cases of PD (Blauwendraat et al., 2019b; Deng et al., 2016; Farlow et al., 2016; Funayama et al., 2015; Guo et al., 2018; Kalia and Lang, 2015; Lesage et al., 2016; Mok et al., 2016; Quadri et al., 2018). Previously, we have replicated *PRKN* (Guo et al., 2008), *PINK1* (Guo et al., 2008; Tang et al., 2006), *PARK7* (Guo et al., 2008; Tang et al., 2006), *ATP13A2* (Guo et al., 2008), *PLA2G6* (Shi et al., 2011), *CHCHD2* (Liu et al., 2015), *RAB39B* (Kang et al., 2016), *TMEM230* (Yan et al., 2017), *GCH1* (Xu et al., 2017), and other genes (Tian et al., 2012; Yang et al., 2019) in patients with PD in China. Copy number variations (CNVs), particularly in *PRKN* and *SNCA*, have also been highlighted to play a vital role in the development of heritable or sporadic PD (Konno et al., 2016; Mok et al., 2016; Taghavi et al., 2018) and several genes, such as *ATP13A2* (Ramirez et al., 2006), *PLA2G6* (Paisan-Ruiz et al., 2009), *FBXO7* (Di Fonzo et al., 2009), *PRKN* (Djarmati et al., 2004; Lucking et al., 2000), *PINK1* (Bonifati et al., 2005; Kumazawa et al., 2008), and *PARK7* (Djarmati et al., 2004) were reported to be strongly associated with an early-onset age of PD (Searles Nielsen et al., 2013).

In addition, genome-wide association studies (GWAS) have provided insight into the genetic basis of PD by identifying and replicating risk loci (Blauwendraat et al., 2019b; Chang et al., 2017; Chung et al., 2012; Foo et al., 2017; Jansen et al., 2017; Nalls et al., 2014). Some differentially DNA methylated genes (Wullner et al., 2016) and differentially expressed genes (Infante et al., 2015) have also been reported to be associated with PD pathogenesis. Most currently known PD-causing genes are involved in several pathogenesis pathways including mitochondrial dysfunction, neuron apoptosis, and the autophagy lysosome ubiquitin-proteasome system (Blauwendraat et al., 2019b). Despite these great advances in the genetics and genomics of PD, the related results are scattered among thousands of studies, making it difficult and laborious for researchers to use these available data.

Parkinson’s disease is an age-dependent neurodegenerative condition, and the age at onset (AAO) is a robust phenotypic measure compared to motor and non-motor phenotypes (Kilarski et al., 2012; Nalls et al., 2015; Wickremaratchi et al., 2011). Many previous studies have reported the frequency of gene mutations in PD patients with different AAOs, and most of them focused on analyzing the association of AAO and several common PD-causing genes (Kasten et al., 2018; Searles Nielsen et al., 2013; Tan et al., 2019; Trinh et al., 2018). Up to now, several genes has been reported to be strongly associated with an early-onset age of PD based on cohorts within specialist clinics or within the general population (Kasten et al., 2018; Lin et al., 2019; Searles Nielsen et al., 2013; Tan et al., 2019; Trinh et al., 2018; Trinh et al., 2019). However, a comprehensive analysis of the association between AAO and all PD-associated genes (PAGs) is still lacking. Furthermore, where AAO is associated with other genetic components, such as the expression pattern, the functional effects of genetic variants need to be further investigated.

To overcome this limitation and facilitate studies of the genetic basis of PD, we integrated multiple layers of genetic and genomic data of PD and prioritized several PAGs, we developed a convenient integrative genomic database named as Gene4PD, which can facilitate queries and analysis of genetic and genomic data with an intuitive graphical user interface that will benefit physicians and researchers.

## Materials and Methods

### Data Collection

We collected five types of genomic data related to PD from the PubMed database with following search items: (1) rare variants identified from PD patients with “Parkinson × [Title/Abstract] AND [mutation (Title/Abstract) or variant (Title/Abstract)] AND gene [Title/Abstract]”; (2) CNVs in PD with “Parkinson × [Title/Abstract] AND [copy number variation (Title/Abstract) or CNV (Title/Abstract)]”; (3) associated SNPs identified from GWAS with “Parkinson × [Title/Abstract] AND GWAS[Title/Abstract]”; (4) differential expressed genes (DEGs) with “Parkinson × [Title/Abstract] AND transcriptome [Title/Abstract]”; (5) differentially expressed DNA methylation genes (DMGs) with “Parkinson × [Title/Abstract] AND DNA methylation [Title/Abstract].” We then screened out articles related to PD according to the exclusion criteria: (i) studies that focused on molecular mechanisms, rather than genetic studies; (ii) studies without original data for genomic information, which may cite from published studies, such as reviews or meta-analysis; and the inclusion criteria: (i) studies reported the genomic information (such as rare variant, SNP, and CNV) of PD, (ii) studies reported the differential expressed genes of PD, (iii) studies reported the differentially expressed DNA methylation genes of PD. Then, we manually extracted available genomic, AAO and other clinical information.

### Data Annotation and PAG Prioritization

To investigate the functional genetic variants identified in patients with PD, ANNOVAR (Wang et al., 2010) was used for comprehensive annotation based on definitions of transcripts from the RefSeq database. Based on the functional effects and minor allele frequency (MAF) in the GnomAD database, the genetic variants were classified as follows: (1) rare loss of function (LoF, including frameshift indels, splicing, stop-gain, and stop-loss) variants with MAF less than 0.01; (2) rare deleterious missense (Dmis) variants with MAF less than 0.01; (3) rare tolerate missense (Tmis) variants with MAF less than 0.01; and (4) all remaining genetic variants. We used our previously developed program ReVe (Ioannidis et al., 2016) to predict deleterious missense variants with scores higher than 0.7, as previous studies.

**FIGURE 1:**
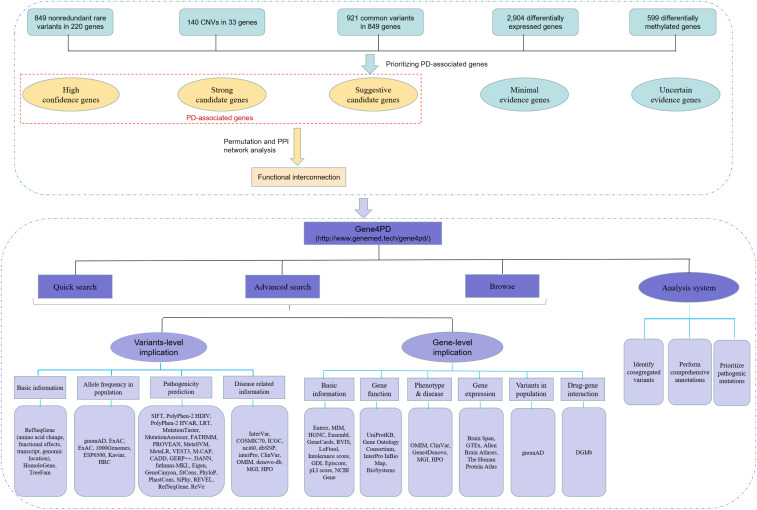
The overall roadmap of this study. The above green dashed box is the process of data collection and analysis, and the below purple dashed box is the overall framework of Gene4PD. Gene4PD supports a “Quick search,” “Advanced search,” “Browse,” and “Analysis” service, as shown in the blue section. The annotation information at the variant level and gene level is shown in the lilac section.

To prioritize PAGs, we developed a scoring system (Supplementary Table 2) combining the different types of genetic evidences as following. The rare variants assigned an evidence scores of 1–5 according to their functional effects and MAF. The CNVs were directly assigned an evidence score of 5 because they disrupted gene function. The PD-associated SNPs, DEGs, and DMGs were assigned an evidence scores of 1–3 based on their *p*-values. A combined evidence score for each gene was calculated by summing up the evidence scores of all integrated studies. All genes integrated in this study were classified into five grades: high confidence (score ≥ 20), strong associated (score of 10–20), suggestive associated (score of 5–10), minimal evidence (score of 3–5), and uncertain evidence (score < 3) (Figure 1).

### The Verification of Functional Correlation and PAG Network

We performed a permutation test to evaluate the interconnectivity and functional correlation among the 124 PAGs (score ≥ 5) as described in our previous study (Li et al., 2017). To ensure a reasonable level of analysis, we constructed a protein-protein interaction (PPI) network using the STRING v 11.0 database^1^ (Szklarczyk et al., 2019) with a confidence score greater than 0.4. Specifically, we randomly simulated 1,000,000 permutation tests to evaluate the interconnectivity among high-confidence/strong genes, and among suggestive genes, as well as to determine the connectivity between these two classes of PAGs. Then, the 124 PAGs were selected to construct an interconnected PPI network based on the STRING online analysis platform. Additionally, the functional networks were clustered by multiple biological processes of Gene Ontology (GO)^2^.

### Association Between Age at Onset and PAGs

The AAO data and genetic data of each gene were downloaded from Gene4PD. We then analyzed the relationship between the AAO and PAGs from three perspectives. First, we aggregated and compared genes with more than five AAO terms to assess the association between AAO and PAGs. All genes were classified into one of three sections: juvenile-onset (≤30 years), early-onset (30–50 years), or late-onset (>50 years), according to the median of the AAO. Second, we compared the AAO with different functional types of variants [loss-of-function (LoF) and deleterious missense variants (Dmis)] in the same gene to investigate the contribution of different types of functional variants (LoF and Dmis) to the AAO of PD.

### Database Construction and Interface

By integrating all the collected genomic information as well as the annotation for each variant/gene from 63 data sources (Supplementary Table 3), we developed *Gene4PD* database^3^ by combining Vue with a PHP-based web framework laravel to construct as a user-friendly web interface. Moreover, the front and back separation model was used for website development. The front end is based on vue and uses the UI Toolkit element, which supports all modern browsers across platforms, including Microsoft Edge, Safari, Firefox, and Google Chrome, and the back end is based on laravel, a PHP web framework. Gene4PD is compatible with all major browser environments and different operating systems such as Windows, Linux, and Mac. The data are stored in the MySQL database.

## Results

### Genomic Data Integration and PAG Prioritization

Through systematic review and curation of multiple lines of public studies, we reviewed more than 3,000 publications, 487 of which met the quality control and collection criteria. Genetic information such as gene symbols, chromosomes, locations, reference base, altered base, and hereditary modes were collected, and other basic information and clinical data, including sample ID, PubMed ID, methods of detecting variants, country, race, gender, AAO, functional study, subtype of disease, detail description of clinical phenotypes, and sporadic/familiar types, were also integrated. We catalogd five types of genetic data (8302 terms) related to PD: (1) 2,252 rare variants, including 954 non-redundant rare variants, in 226 genes from 327 available publications; (2) 139 CNVs in 34 genes from 94 publications; (3) 1,237 associated SNPs in 640 genes from 42 studies; (4) 2,926 DEGs from 8 publications; (5) 657 DMGs from 7 publications (Supplementary Table 1) (Figure 1). Specifically, for the collected genetic variants, we identified 334 rare LoF, 1,328 rare Dmis variants, 485 rare Tmis variants, and 105 other remaining variants based on standard annotations.

We then developed a weighted scoring system to prioritize PAGs by combining all above genetic evidence. As a result, we prioritized 25 high confidence genes (score ≥ 20), 38 strong associated genes (score of 10–20), 61 suggestive associated genes (score of 5–10), 88 minimal evidence genes (score of 3–5), and 3,791 uncertain evidence genes (score < 3) (Table 1). We found that 19 of 21 known PD-causing genes (Supplementary Table 4) were prioritized as high confidence genes. Six other high confidence genes, including *GCH1*, *RAB39B*, *CHCHD2*, *MAPT*, *TH*, and *ASNA1*, were also regarded as potential PD-causing genes. The remaining two known PD-causing genes were classified into strong associated genes (*HTRA2*) or suggestive associated genes (*UCHL1*), as they showed lower replication.

**TABLE 1:** Prioritized associated genes in Gene4PD.

**High confidence genes (score ≥ 20) (*N* = 25)**	**Strong associated genes (score of 10–20) (*N* = 19)**	**Suggestive associated genes (score of 5–10) (*N* = 66)**
PRKN^#^, PINK1^#^, LRRK2^#^, SNCA^#^, GBA^#^, PARK7^#^, GCH1, PLA2G6^#^, ATP13A2^#^, VPS35^#^, DNAJC13^#^, FBXO7^#^, POLG^#^, TMEM230^#^, SYNJ1^#^, GIGYF2^#^, VPS13C^#^, LRP10^#^, RAB39B, CHCHD2, MAPT, TH, EIF4G1^#^, ASNA1, and DNAJC6^#^	TNR, PODXL, CSMD1, HTRA2^#^, GPRIN3, PPM1K, TARDBP, SLC6A3, ATP10B, PTEN, MMRN1, VAPB, L2HGDH, SPP1, SCN3A, NAP1L5, ITPR1, PZP, UQCRC1, DCTN1, ABCG2, ATOH1, CCSER1, FAM13A, FAM13A-AS1, GRID2, HERC3, HERC5, HERC6, PIGY, PKD2, PYURF, SMARCAD1, TIGD2, ANKRD30A, DIS3, MNS1, and PTRHD1	RIC3, SLC18A2, COL6A5, ATP1A3, CD36, CP, GRN, PSEN1, SMPD1, FAM83A, KIF21A, PTPRH, COMT, SPG7, MCCC1, PLIN4, TNK2, UCHL1^#^, APOE, OGN, WDR45, TBC1D24, TWNK, BST1, CAPS2, CEL, SVOPL, ATG4C, CABIN1, COL15A1, DARS, DNAH8, ELOA2, FAM71A, FAM90A1, FER1L6, GH2, GPATCH2L, GRAMD1C, IFI35, KALRN, KCNK16, LIPI, LPA, MAP3K6, MS4A5, NUS1, OR8B3, PCDHA9, PRB3, PRMT3, PRSS48, PTCHD3, RFPL2, SCARF2, SPPL2C, TMEM134, UHRF1BP1L, USP20, ZNF516, and ZNF543

*^#^Known PD-causing genes [Blauwendraat et al. (2019b). The genetic architecture of Parkinson’s disease. *Lancet Neurol*].124 PD-associated genes (score ≥ 5) were classified into three grades, including high confidence (score ≥ 20), strong associated (score of 10–20), and suggestive associated (score of 5–10).*

### PAGs Were Functionally Correlated

We performed a permutation test to assess the functional correlations of the 124 associated genes (score ≥ 5). Most of them were disease-causing genes or risk-genes from GWAS. As a result, we observed 48 of 63 high-confidence or strong PAGs (*P* < 1.0 × 10^–6^, Supplementary Figure 1A) that interacted with each other and had 203 interconnections (*P* < 1.0 × 10^–6^, Supplementary Figure 1B), which was significantly higher than the random expectation. Similarly, the suggestive associated genes also significantly interacted with each other (Supplementary Figures 1C,D), suggesting they were functionally correlated. Furthermore, we observed 21 suggestive associated genes (*P* = 0.098, Supplementary Figure 1E) which interacted with high-confidence or strong PAGs with 74 connections (*P* = 2.5 × 10^–5^, Supplementary Figure 1F), suggesting that the two classes of associated genes were also functionally correlated. These results demonstrate that the 124 genes were functionally associated with PD, although these results require further experimental validation.

**FIGURE 2:**
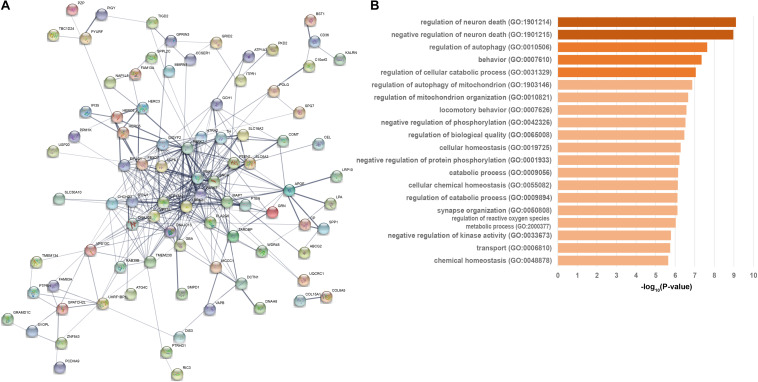
Functional network and biological progresses of Parkinson’s disease-associated genes. **(A)** Functional network of closely related Parkinson’s disease-associated genes based on the STRING database. Nodes are colored to show the associations, and the thickness of lines connecting nodes indicates the strength of the association between nodes. **(B)** Biological progresses of gene ontology (GO) terms are involved in functional PPI network this network. The heavier the bar color is, the P-value more significant is.

We then developed an interacted functional network contained 88 of the 124 PAGs which interacted with each other at protein-level with 336 connections (Figure 2A). The functional network contained 25 high-confidence genes, 27 strong associated genes, and 36 suggestive associated genes. All 21 known PD-causing genes were included in this functional network. Additionally, other genes in this PPI network may be associated with PD. GO enrichment analysis of the 88 genes revealed server GO-associated with PD (Supplementary Table 5 and Figure 2B). Most of these GO terms, such as regulation of neuron death (GO:1901214, FDR = 7.81 × 10^–10^), regulation of autophagy (GO:0010506, FDR = 2.30 × 10^–8^), behavior (GO:0007610, FDR = 4.46 × 10^–8^), regulation of cellular catabolic process (GO:0031329, FDR = 8.84 × 10^–8^) (Supplementary Table 5 and Figure 2B), were regarded as critical functional signaling pathways associated with PD. The functional network suggested that the prioritized PAGs shared a common signaling mechanism and were functionally correlated.

### AAO Is Associated With Multiple Genetic Components

**FIGURE 3:**
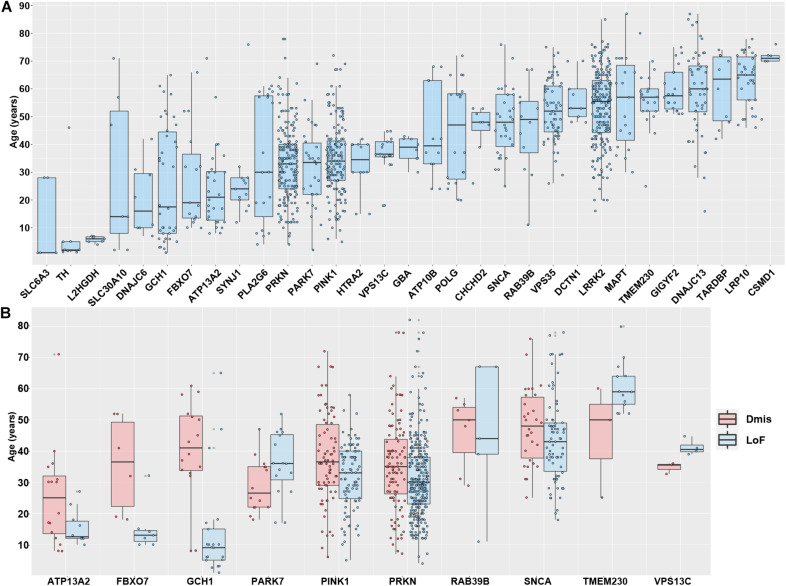
Association between Parkinson’s disease-associated genes and age at onset. **(A)** The association between age at onset (AAO) and 31 Parkinson’s disease-associated genes with more than five AAO items. The genes shown are sorted according to the median AAO or each gene. **(B)** Association between AAO and 10 Parkinson’s disease-associated genes with more three loss-of-function (LoF) variants and deleterious missense (Dmis) variants.

The integrated genetic data and AAO data from the Gene4PD database provide an unprecedented opportunity to comprehensively identify the vital association between the AAO and PAGs on a large scale. Therefore, we analyzed 31 PAGs with more than five AAO items in each gene (Figure 3A). After sorting by the median AAO of each gene, we found that 10 genes (*SLC6A3, TH, L2HGDH, SLC30A10, DNAJC6*, *GCH1*, *FBXO7*, *ATP13A2*, *SYNJ1*, and *PLA2G6*) were associated with a juvenile-onset (age ≤ 30 years), 11 genes (*PRKN*, *PARK7*, *PINK1*, *HTRA2*, *VPS13C, GBA, ATP10B, POLG*, *CHCHD2*, *SNCA*, *and RAB39B*) were associated with an early-onset (age of 30–50 years), whereas another 10 genes (*VPS35*, *DCTN1*, *LRRK2*, *MAPT, TMEM230*, *GIGYF2*, *DNAJC13*, *TARDBP*, *LRP10*, *and CSMD1*) were associated with a late-onset (age > 50 years). Although different PAGs had specific AAO characteristics, we noted that there were large differences in the AAO for some genes, such as for *PRKN*, *PINK1*, *SNCA*, and *LRRK2*, suggesting that there are other factors that contribute to the AAO.

To further investigate different types of functional variants (LoF and Dmis) contributing to the AAO of PD, we further performed pairwise comparison to analyze the differences in the AAO between patients with LoF and patients with Dmis for each gene. As a result, 10 genes with more than three AAO items both in LoF and Dmis were selected to perform a comparison analysis. We found that the AAO of patients with LoF in five genes, including *GCH1* (*P* = 2.63 × 10^–5^), *PINK1* (*P* = 2.31 × 10^–3^), *PRKN* (*P* = 4.08 × 10^–3^), *FBXO7* (*P* = 0.02), and *ATP13A2* (*P* = 0.03), were significantly lower than patients with Dmis, while the AAO of patients with LoF in *VPS13C* (*P* = 0.01) was higher than patients with Dmis, whereas four other genes including *PARK7* (*P* = 0.06), *SNCA* (*P* = 0.12), *RAB39B* (*P* = 0.96), and *TMEM230* (*P* = 0.27) did not present significant differences (Figure 3B). More PD samples with detailed genotypes and phenotypes may be identified in further studies investigating the relationship between AAO and PAGs.

### Gene4PD: An Integrative Genetic Database and Analytic Platform for Parkinson’s Disease

**FIGURE 4:**
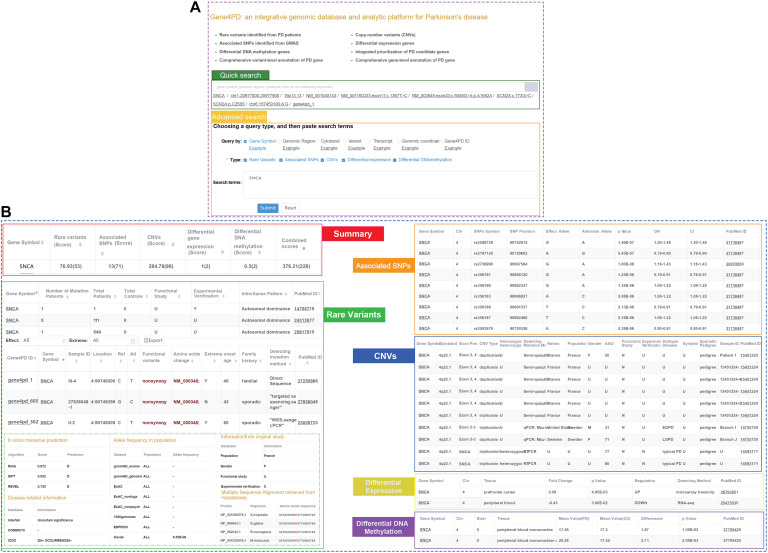
Snapshot of search panel and variant-level implications in Gene4PD. **(A)** Snapshot of search panel in Gene4PD. Quick search panel and advanced search panel in Gene4PD are illustrated. **(B)** Snapshot of variant-level implications in Gene4PD. Six sections including “Summary,” “Rare variant,” “Associated SNPs,” “CNVs,” “Differential expression gene,” “Differential DNA methylation,” are set in variant-level implication.

To facilitate researchers to query and analyze genetic and genomic data related to PD, we constructed an online comprehensive database and analysis platform named as Gene4PD^4^ which integrated rare variants, CNVs, associated SNPs, DEGs, and DMGs related PD and more than 63 popular genomic and genetic data sources (Figure 1 and Supplementary Table 3). Users can query the detailed genetic and genomic information of the variants and genes *via* quick or advanced search interfaces in Gene4PD (Figure 4A). The quick search supports several common search terms (such as gene symbol, genomic regions, cytoband) and returns a page containing six visual tables. The advanced search in Gene4PD supports additional user demands by allowing the user to upload a file or paste a list containing search terms. The first table summarizes the evidences scores from the variant types of the collected genetic data, and the other five tables show the more detailed information (Figure 4B). In addition, to comprehensively evaluate the pathogenicity of genetic variants, three meaningful panels were integrated in Gene4PD: (1) predicted damaging scores and functional consequences of missense variants from 24 *in silico* algorithms (Supplementary Table 3); (2) allele frequencies of different populations based on seven databases, including gnomAD (Lek et al., 2016), ExAC (Karczewski et al., 2017; Lek et al., 2016), 1000 Genomes Project (Genomes Project et al., 2015), ESP6500 (Fu et al., 2013), Kaviar, and Haplotype Reference Consortium; (3) disease-related information from 11 related databases, including InterVar (Li and Wang, 2017), COSMIC70 (Forbes et al., 2017), ICGC (International Cancer Genome Consortium, Hudson et al., 2010), dbSNP (Sherry et al., 2001), ClinVar (Landrum et al., 2020), Gene4Denovo (Guihu et al., 2019), InterPro (Finn et al., 2017), OMIM (Amberger et al., 2019), MGI (Eppig et al., 2017), and HPO (Kohler et al., 2020). Notably, all query results can be copied to the clipboard or exported as Excel spreadsheets.

Gene4PD includes six sections to obtain gene-level knowledge of a given gene in a one-stop interface (Supplementary Figure 2). (1) The Basic information sections gives out the primary information of genes sourced from NCBI Gene, Entrez gene, Ensembl, OMIM, GeneCards, HGNC, the intolerance score from residual variation intolerance score (RVIS), LoFtool, heptanucleotide context intolerance score, GDI, Episcore, and pLI score; (2) The Gene function section includes five sub-sections: molecular function extracted from UniportKB, GO terms, Domian from InterPro, PPI from InBio Map, biological pathway from Biosystems; (3) The Phenotype and Disease section reports phenotype and disease-related variants or genes from the OMIM (Amberger et al., 2019), ClinVar (Landrum et al., 2020), MGI (Eppig et al., 2017), HPO (Kohler et al., 2020) and Gene4Denovo (Guihu et al., 2019) are shown in the Phenotype and disease section; (4) The Gene expression section shows spatiotemporal-expression levels of genes in the brain retrieved from BrianSpan, expression profiles in 31 primary tissues and in 54 secondary tissues extracted from GTEx, cell diversity and expression in the human cortex based on single-nucleus RNA-seq data from Allen Brain Atlas, and the subcellular location retrieved from The Human Protein Atlas; (5) The Variants in different populations section provides the number of variants with functional effects in different populations from genomAD; (6) The Drug-gene interaction section provides the drug-gene interactions and gene druggability data sourced from DGIdb v3.0.2.

One of the main advantages of Gene4PD is that it provides an interface for analyzing genetic data according to the specific needs of the user (Supplementary Figure 3). The analysis process includes four simple steps of filling in an email address, choosing the Trio or Non-trio option of the genetic data, uploading genetic data files (VCF4 format), and inputting basic information of samples. Gene4PD will then analyze the rare damaging variants using default parameters. Importantly, Gene4PD also supports a flexible control panel so that users can conveniently adjust the values of quality control, data sources of annotation, and parameters for identifying rare damaging variants. The annotation section contains four sub-panels: Basic information annotation, Pathogenicity prediction of missense variants, Allele frequency in variant population, and Clinical-related database. When the analysis is complete, Gene4PD will send a link to the user by email for downloading the results.

## Discussion

With the development of NGS, the number of multi-omics studies has greatly increased, such as genomics, genetics, epigenetics, and transcriptomics, for revealing the pathogenesis of PD, resulting in the identification of numerous associated genes (Jansen et al., 2017; Sandor et al., 2017; Siitonen et al., 2017). The ability to comprehensively investigate these associated genes from different genetic perspectives such as expression patterns, functional interconnections, and relationships with AAO would be helpful for studying the pathogenesis of PD, and improving clinical diagnosis, treatment, and drug development efforts (Blauwendraat et al., 2019b). However, most of these results are widely scattered among published articles, making it challenging to integrate these omics data. To facilitate this task, in this study, we manually collected rare variants, CNVs, associated SNPs, DEGs, and DMGs related to PD from the related literature, and prioritized 124 PAGs, including 25 high-confidence, 38 strong associated and 61 suggestive associated genes. Interestingly, 19 of the 21 known PD-causing genes belonged to the high-confidence genes, highlighting that other high-confidence PAGs may also be associated with PD. Therefore, more genetic and experimental studies are needed to validate the genetic mechanisms of PAGs incorporating all of these associations.

We further performed several bioinformatics analyses to validate the functional association of the 124 PAGs based on PPI data. First, we observed significant associations among the high-confidence/strong PAGs, among the suggestive PAGs, and between the two classes of experimental evidences. Second, we developed an interconnected functional network containing 88 PAGs and enriched in several known PD-associated functional pathways. In the functional network, we highlighted several PAGs that interacted with known PD-causing genes, such as *CHCHD2*, *RAB39B*, and *RIC3. CHCHD2* (Funayama et al., 2015) has been widely reported to be associated with PD, presented functional interaction with many PD-causing genes, such as *SNCA*, *PINK1*, *LRRK2*, *PARK7*, *VPS35* in the network. *RAB39B* with the known PD-causing genes *SNCA* and *LRRK2* is consistent with its α-synuclein pathology (Wilson et al., 2014). These results suggest that these three genes are involved in closely related biological functions similar to the disease-causing genes and increasing the risk PD.

To enable PD researchers to make full use of the reported genetic data, more than six online PD-related database such as the variation databases MDSGene (Lill et al., 2016), PDGene database (Nalls et al., 2014), ParkDB (Taccioli et al., 2011), Parkinson Disease Mutation Database (PDmutDB) (Horaitis et al., 2007), The Mutation Database for PD (MDPD) (Tang et al., 2009), (Parkinson’s disease map) PDMap (Fujita et al., 2014) and PDbase (Yang et al., 2009) have been explored. However, we found that MDSGene collected mutations of 12 common PD associated genes, and the other three databases (PDmutDB, MDPD, PDbase) were not in functional order for use. In addition, the PDGene database focused on GWAS data collection and analysis and did not provide summary and statistic reports, whereas ParkDB is specifically dedicated to gene expression data and PDmap integrates pathways implicated in PD pathogenesis. Furthermore, there are also other human disease-related genetic databases, which are not specially integrated PD-related genetic information, including DisGeNet{Pinero, 2020 #226} and Open Targets Platform{Ochoa, 2020 #225}. Therefore, to establish an integrative genetic database and analytic platform for facilitating PD research, we integrated all collected data to construct Gene4PD as a comprehensive data analysis platform. Compared to existing databases, Gene4PD not only integrates more recorded variants, CNVs, SNPs from GWAS, DNA methylation genes, and DEGs, but also supports the systematic analysis of genetic variants. To overcome the multiple challenges in evaluating the pathogenicity of a variant or to determine whether a gene is pathogenic, based on experience with the construction of our previous online databases VarCards (Li et al., 2018) and Gene4Denovo (Guihu et al., 2019), we integrated more than 63 genomic data resources in Gene4PD, covering comprehensive variant-level and gene-level annotation data. In addition, we will update Gene4PD by collecting published gene-related data every 6 months.

PD has been widely recognized as an age-dependent neurodegenerative condition. The variation in phenotypes and genotypes can be related to the AAO of PD (Kilarski et al., 2012; Nalls et al., 2015; Wickremaratchi et al., 2011). Previous studies suggested that some genes were strongly associated with the AAO, such as *ATP13A2* (Ramirez et al., 2006), *PLA2G6* (Paisan-Ruiz et al., 2009), *FBXO7* (Di Fonzo et al., 2009), *PRKN* (Djarmati et al., 2004; Lucking et al., 2000), *PINK1* (Bonifati et al., 2005; Kumazawa et al., 2008), and *PARK7* (Djarmati et al., 2004). Based on our largescale integrated dataset, we not only confirmed the association between these six commonly reported genes and AAO, but also verified another 20 genes associated with AAO. Furthermore, our results suggest that different types of functional variants (LoF and Dmis) contribute differently to the AAO of PD.

There were some limitations to this study. First, by satisfying the quality control, we collected as much related literature as possible. Although all data collectors, who were rigorously trained to ensure the consistency of collected data, were researchers or PhD students with a strong background in clinical genetics, there may have been some omissions. We encourage researchers to contact us to refine missing data of PD. We will keep to update it annually. Second, the scoring system used in this study may not be powerful enough, and thus we encourage users to analyze genetic data with the re-score function in Gene4PD or to download data for re-analysis. Third, the integrated approach in this study may be biased, and the prioritized genes need to be validated in population and verified the pathogenic mechanism with cell or animal experiments. Fourth, we did not get much data on AAO of PD. We hope to collect more AAO data in the future to make our analysis results to be more reliable. Last, clinical information besides the AAO must be considered to determine the factors contributing to the genotype-phenotype correlations of PD. However, most articles do not provide detailed phenotypic information, which poses a challenge for a meta-analysis of the relationship between clinical phenotypes and genes. In this regard, we encourage researchers to provide details on clinical phenotypes in the context of genetic studies.

## Conclusion

In conclusion, we catalogd different types of genetic data from many publications related to PD and prioritized 124 functionally related PAGs with different lines of evidence, which were used to construct the database and analysis tool Gene4PD, suggesting that integrating multiple genetic data is useful for prioritizing novel associated genes. In addition, we characterized the genetic landscape of the prioritized PAGs, providing insight into the pathology of PD. Gene4PD is expected to provide researchers and clinicians comprehensive genetic knowledge and analytic platform for PD, and would also improve the understanding of pathogenesis in PD.

## Data Availability Statement

Publicly available datasets were analyzed in this study. This data can be found here: http://genemed.tech/gene4pd/.

## Author Contributions

BL, GZ, BT, and JL: study design. BL, GZ, QZ, YX, ZW, ZF, BLu, LQ, YWZ, RZ, LJ, HP, YH, XW, TL, YZ, YW, QC, and JL: literature search and data collection. BL, GZ, QZ, YX, BT, and JL: figures. BL, GZ, ZF, BLu, XW, TL, YZ, ZL, JG, and JL: data analysis. BL, GZ, ZW, YW, QC, BT, and JL: data interpretation. BL, GZ, QZ, YX, ZW, ZF, BLu, LQ, YWZ, RZ, LJ, HP, YH, XW, TL, YZ, YW, QC, ZL, JG, BT, and JL: writing of the manuscript draft and all revision stages. All authors contributed to the article and approved the submitted version.

## Conflict of Interest

The authors declare that the research was conducted in the absence of any commercial or financial relationships that could be construed as a potential conflict of interest.

## Footnotes

1 https://string-db.org/

2 http://www.geneontology.org/

3 http://www.genemed.tech/gene4pd/

4 http://www.genemed.tech/gene4pd/

## References

[B1] Alzheimer’s Association. (2014). 2014 Alzheimer’s disease facts and figures. *Alzheimers Dement.* 10 e47–92. 10.1016/j.jalz.2014.02.001 24818261

[B2] Amberger J. S. Bocchini C. A. Scott A. F. Hamosh A. (2019). OMIM.org: leveraging knowledge across phenotype-gene relationships. *Nucleic Acids Res.* 47 D1038–D1043. 30445645 10.1093/nar/gky1151 PMC6323937

[B3] Blauwendraat C. Heilbron K. Vallerga C. L. Bandres-Ciga S. von Coelln R. Pihlstrom L. (2019a). Parkinson’s disease age at onset genome-wide association study: defining heritability, genetic loci, and alpha-synuclein mechanisms. *Mov. Disord.* 34 866–875. 30957308 10.1002/mds.27659 PMC6579628

[B4] Blauwendraat C. Nalls M. A. Singleton A. B. (2019b). The genetic architecture of Parkinson’s disease. *Lancet Neurol.* 19 170–178. 31521533 10.1016/S1474-4422(19)30287-X PMC8972299

[B5] Bonifati V. Rohe C. F. Breedveld G. J. Fabrizio E. De Mari M. Tassorelli C. (2005). Early-onset parkinsonism associated with PINK1 mutations: frequency, genotypes, and phenotypes. *Neurology* 65 87–95. 10.1212/01.wnl.0000167546.39375.82 16009891

[B6] Chang D. Nalls M. A. Hallgrimsdottir I. B. Hunkapiller J. van der Brug M. Cai F. (2017). A meta-analysis of genome-wide association studies identifies 17 new Parkinson’s disease risk loci. *Nat. Genet.* 49 1511–1516. 28892059 10.1038/ng.3955 PMC5812477

[B7] Chung S. J. Armasu S. M. Biernacka J. M. Anderson K. J. Lesnick T. G. Rider D. N. (2012). Genomic determinants of motor and cognitive outcomes in Parkinson’s disease. *Parkinsonism Relat. Disord.* 18 881–886. 10.1016/j.parkreldis.2012.04.025 22658654 PMC3606821

[B8] Deng H. X. Shi Y. Yang Y. Ahmeti K. B. Miller N. Huang C. (2016). Identification of TMEM230 mutations in familial Parkinson’s disease. *Nat. Genet.* 48 733–739. 27270108 10.1038/ng.3589 PMC6047531

[B9] Di Fonzo A. Dekker M. C. Montagna P. Baruzzi A. Yonova E. H. Correia Guedes L. (2009). FBXO7 mutations cause autosomal recessive, early-onset parkinsonian-pyramidal syndrome. *Neurology* 72 240–245. 10.1212/01.wnl.0000338144.10967.2b 19038853

[B10] Djarmati A. Hedrich K. Svetel M. Schafer N. Juric V. Vukosavic S. (2004). Detection of Parkin (PARK2) and DJ1 (PARK7) mutations in early-onset Parkinson disease: parkin mutation frequency depends on ethnic origin of patients. *Hum. Mutat.* 23:525. 10.1002/humu.9240 15108293

[B11] Dorsey E. R. Constantinescu R. Thompson J. P. Biglan K. M. Holloway R. G. Kieburtz K. (2007). Tanner, Projected number of people with Parkinson disease in the most populous nations, 2005 through 2030. *Neurology* 68 384–386. 10.1212/01.wnl.0000247740.47667.03 17082464

[B12] Eppig J. T. Smith C. L. Blake J. A. Ringwald M. Kadin J. A. Richardson J. E. (2017). Mouse Genome Informatics (MGI): resources for Mining Mouse Genetic, Genomic, and Biological Data in Support of Primary and Translational Research. *Methods Mol. Biol.* 1488 47–73. 10.1007/978-1-4939-6427-7_3 27933520

[B13] Farlow J. L. Robak L. A. Hetrick K. Bowling K. Boerwinkle E. Coban-Akdemir Z. H. (2016). Whole-Exome Sequencing in Familial Parkinson Disease. *JAMA Neurol.* 73 68–75. 26595808 10.1001/jamaneurol.2015.3266 PMC4946647

[B14] Finn R. D. Attwood T. K. Babbitt P. C. Bateman A. Bork P. Bridge A. J. (2017). InterPro in 2017-beyond protein family and domain annotations. *Nucleic Acids Res.* 45 D190–D199. 27899635 10.1093/nar/gkw1107 PMC5210578

[B15] Foo J. N. Tan L. C. Irwan I. D. Au W. L. Low H. Q. Prakash K. M. (2017). Genome-wide association study of Parkinson’s disease in East Asians. *Hum. Mol. Genet.* 26 226–232. 28011712 10.1093/hmg/ddw379

[B16] Forbes S. A. Beare D. Boutselakis H. Bamford S. Bindal N. Tate J. (2017). COSMIC: somatic cancer genetics at high-resolution. *Nucleic Acids Res.* 45 D777–D783. 27899578 10.1093/nar/gkw1121 PMC5210583

[B17] Fu W. O’Connor T. D. Jun G. Kang H. M. Abecasis G. Leal S. M. (2013). Analysis of 6,515 exomes reveals the recent origin of most human protein-coding variants. *Nature* 493 216–220. 10.1038/nature11690 23201682 PMC3676746

[B18] Fujita K. A. Ostaszewski M. Matsuoka Y. Ghosh S. Glaab E. Trefois C. (2014). Integrating pathways of Parkinson’s disease in a molecular interaction map. *Mol. Neurobiol.* 49 88–102. 10.1007/s12035-013-8489-4 23832570 PMC4153395

[B19] Funayama M. Ohe K. Amo T. Furuya N. Yamaguchi J. Saiki S. (2015). CHCHD2 mutations in autosomal dominant late-onset Parkinson’s disease: a genome-wide linkage and sequencing study. *Lancet Neurol.* 14 274–282. 10.1016/s1474-4422(14)70266-2 25662902

[B20] Genomes Project C. Auton A. Brooks L. D. Durbin R. M. Garrison E. P. Kang H. M. (2015). A global reference for human genetic variation. *Nature* 526 68–74. 10.1038/nature15393 26432245 PMC4750478

[B21] Guihu Z. Kuokuo L. Bin L. Zheng W. Zhenghuan F. Xiaomeng W. (2019). Gene4Denovo: an integrated database and analytic platform for de novo mutations in humans. *Nucleic Acids Res.* 48 D913–D926. 10.1093/nar/gkz923 PMC7145562 31642496

[B22] Guo J. F. Xiao B. Liao B. Zhang X. W. Nie L. L. Zhang Y. H. (2008). Mutation analysis of Parkin, PINK1, DJ-1 and ATP13A2 genes in Chinese patients with autosomal recessive early-onset Parkinsonism. *Mov. Disord.* 23 2074–2079. 10.1002/mds.22156 18785233

[B23] Guo J. F. Zhang L. Li K. Mei J. P. Xue J. Chen J. (2018). Coding mutations in NUS1 contribute to Parkinson’s disease. *Proc. Natl. Acad. Sci. U. S. A.* 115 11567–11572. 30348779 10.1073/pnas.1809969115 PMC6233099

[B24] Horaitis O. Talbot C. C. Jr. Phommarinh M. Phillips K. M. Cotton R. G. database A. (2007). of locus-specific databases. *Nat. Genet.* 39:425. 10.1038/ng0407-425 17392794

[B25] Infante J. Prieto C. Sierra M. Sanchez-Juan P. Gonzalez-Aramburu I. Sanchez-Quintana C. (2015). Identification of candidate genes for Parkinson’s disease through blood transcriptome analysis in LRRK2-G2019S carriers, idiopathic cases, and controls. *Neurobiol. Aging* 36 1105–1109. 10.1016/j.neurobiolaging.2014.10.039 25475535

[B26] International Cancer Genome Consortium, Hudson T. J. Anderson W. Artez A. Barker A. D. Bell C. (2010). International network of cancer genome projects. *Nature* 464 993–998. 10.1038/nature08987 20393554 PMC2902243

[B27] Ioannidis N. M. Rothstein J. H. Pejaver V. Middha S. McDonnell S. K. Baheti S. (2016). REVEL: an Ensemble Method for Predicting the Pathogenicity of Rare Missense Variants. *Am. J. Hum. Genet.* 99 877–885. 27666373 10.1016/j.ajhg.2016.08.016 PMC5065685

[B28] Jansen I. E. Ye H. Heetveld S. Lechler M. C. Michels H. Seinstra R. I. (2017). Discovery and functional prioritization of Parkinson’s disease candidate genes from large-scale whole exome sequencing. *Genome Biol.* 18:22. 10.1186/s13059-017-1147-9 PMC5282828 28137300

[B29] Kalia L. V. Lang A. E. (2015). Parkinson’s disease. *Lancet* 386 896–912. 25904081 10.1016/S0140-6736(14)61393-3

[B30] Kang J. F. Luo Y. Tang B. S. Wan C. M. Yang Y. Li K. (2016). RAB39B gene mutations are not linked to familial Parkinson’s disease in China. *Sci. Rep.* 6:34502. 10.1038/srep34502 PMC5046083 27694831

[B31] Karczewski K. J. Weisburd B. Thomas B. Solomonson M. Ruderfer D. M. Kavanagh D. (2017). The ExAC browser: displaying reference data information from over 60 000 exomes. *Nucleic Acids Res.* 45 D840–D845. 27899611 10.1093/nar/gkw971 PMC5210650

[B32] Kasten M. Hartmann C. Hampf J. Schaake S. Westenberger A. Vollstedt E. J. (2018). Genotype-Phenotype Relations for the Parkinson’s Disease Genes Parkin, PINK1, DJ1: MDSGene Systematic Review. *Mov. Disord.* 33 730–741. 10.1002/mds.27352 29644727

[B33] Kilarski L. L. Pearson J. P. Newsway V. Majounie E. Knipe M. D. Misbahuddin A. (2012). Systematic review and UK-based study of PARK2 (parkin), PINK1, PARK7 (DJ-1) and LRRK2 in early-onset Parkinson’s disease. *Mov. Disord.* 27 1522–1529. 10.1002/mds.25132 22956510

[B34] Kohler S. Gargano M. Matentzoglu N. Carmody L. C. Lewis-Smith D. Vasilevsky N. A. (2020). The Human Phenotype Ontology in 2021. *Nucleic Acids Res.* 49 D1207–D1217. 10.1093/nar/gkaa1043 PMC7778952 33264411

[B35] Konno T. Ross O. A. Puschmann A. Dickson D. W. Wszolek Z. K. (2016). Autosomal dominant Parkinson’s disease caused by SNCA duplications. *Parkinsonism Relat. Disord.* 22 S1–6. 26350119 10.1016/j.parkreldis.2015.09.007 PMC4820832

[B36] Kumazawa R. Tomiyama H. Li Y. Imamichi Y. Funayama M. Yoshino H. (2008). Mutation analysis of the PINK1 gene in 391 patients with Parkinson disease. *Arch. Neurol.* 65 802–808. 18541801 10.1001/archneur.65.6.802

[B37] Labbe C. Lorenzo-Betancor O. Ross O. A. (2016). Epigenetic regulation in Parkinson’s disease. *Acta Neuropathol.* 132 515–530. 27358065 10.1007/s00401-016-1590-9 PMC5026906

[B38] Landrum M. J. Chitipiralla S. Brown G. R. Chen C. Gu B. Hart J. (2020). ClinVar: improvements to accessing data. *Nucleic Acids Res.* 48 D835–D844. 31777943 10.1093/nar/gkz972 PMC6943040

[B39] Lek M. Karczewski K. J. Minikel E. V. Samocha K. E. Banks E. Fennell T. (2016). Analysis of protein-coding genetic variation in 60,706 humans. *Nature* 536 285–291. 27535533 10.1038/nature19057 PMC5018207

[B40] Lesage S. Drouet V. Majounie E. Deramecourt V. Jacoupy M. Nicolas A. (2016). Loss of VPS13C Function in Autosomal-Recessive Parkinsonism Causes Mitochondrial Dysfunction and Increases PINK1/Parkin-Dependent Mitophagy. *Am. J. Hum. Genet.* 98 500–513. 26942284 10.1016/j.ajhg.2016.01.014 PMC4800038

[B41] Li J. Shi L. Zhang K. Zhang Y. Hu S. Zhao T. (2018). VarCards: an integrated genetic and clinical database for coding variants in the human genome. *Nucleic Acids Res.* 46 D1039–D1048. 29112736 10.1093/nar/gkx1039 PMC5753295

[B42] Li J. Wang L. Guo H. Shi L. Zhang K. Tang M. (2017). Targeted sequencing and functional analysis reveal brain-size-related genes and their networks in autism spectrum disorders. *Mol. Psychiatry* 22 1282–1290. 10.1038/mp.2017.140 28831199

[B43] Li Q. Wang K. (2017). InterVar: clinical Interpretation of Genetic Variants by the 2015 ACMG-AMP Guidelines. *Am. J. Hum. Genet.* 100 267–280. 10.1016/j.ajhg.2017.01.004 28132688 PMC5294755

[B44] Lill C. M. Mashychev A. Hartmann C. Lohmann K. Marras C. Lang A. E. (2016). Launching the movement disorders society genetic mutation database (MDSGene). *Mov. Disord.* 31 607–609. 10.1002/mds.26651 27156390

[B45] Lin C. H. Chen P. L. Tai C. H. Lin H. I. Chen C. S. Chen M. L. (2019). A clinical and genetic study of early-onset and familial parkinsonism in taiwan: an integrated approach combining gene dosage analysis and next-generation sequencing. *Mov. Disord.* 34 506–515. 10.1002/mds.27633 30788857 PMC6594087

[B46] Liu Z. Guo J. Li K. Qin L. Kang J. Shu L. (2015). Mutation analysis of CHCHD2 gene in Chinese familial Parkinson’s disease. *Neurobiol. Aging* 36 3117.e7–3117.e8. 10.1016/j.neurobiolaging.2015.08.010 26343503

[B47] Lucking C. B. Durr A. Bonifati V. Vaughan J. De Michele G. Gasser T. (2000). Association between early-onset Parkinson’s disease and mutations in the parkin gene. *N. Engl. J. Med.* 342 1560–1567. 10824074 10.1056/NEJM200005253422103

[B48] Mok K. Y. Sheerin U. Simon-Sanchez J. Salaka A. Chester L. Escott-Price V. (2016). Deletions at 22q11.2 in idiopathic Parkinson’s disease: a combined analysis of genome-wide association data. *Lancet Neurol.* 15 585–596. 27017469 10.1016/S1474-4422(16)00071-5 PMC4828586

[B49] Nalls M. A. Escott-Price V. Williams N. M. Lubbe S. Keller M. F. Morris H. R. (2015). Disease Genomics, Genetic risk and age in Parkinson’s disease: continuum not stratum. *Mov. Disord.* 30 850–854. 10.1002/mds.26192 25778492 PMC5217457

[B50] Nalls M. A. Pankratz N. Lill C. M. Do C. B. Hernandez D. G. Saad M. (2014). Large-scale meta-analysis of genome-wide association data identifies six new risk loci for Parkinson’s disease. *Nat. Genet.* 46 989–993. 25064009 10.1038/ng.3043 PMC4146673

[B51] Paisan-Ruiz C. Bhatia K. P. Li A. Hernandez D. Davis M. Wood N. W. (2009). Characterization of PLA2G6 as a locus for dystonia-parkinsonism. *Ann. Neurol.* 65 19–23. 10.1002/ana.21415 18570303 PMC9016626

[B52] Quadri M. Mandemakers W. Grochowska M. M. Masius R. Geut H. Fabrizio E. (2018). LRP10 genetic variants in familial Parkinson’s disease and dementia with Lewy bodies: a genome-wide linkage and sequencing study. *Lancet Neurol.* 17 597–608. 29887161 10.1016/S1474-4422(18)30179-0

[B53] Ramirez A. Heimbach A. Grundemann J. Stiller B. Hampshire D. Cid L. P. (2006). Hereditary parkinsonism with dementia is caused by mutations in ATP13A2, encoding a lysosomal type 5 P-type ATPase. *Nat. Genet.* 38 1184–1191. 10.1038/ng1884 16964263

[B54] Sandor C. Honti F. Haerty W. Szewczyk-Krolikowski K. Tomlinson P. Evetts S. (2017). Whole-exome sequencing of 228 patients with sporadic Parkinson’s disease. *Sci. Rep.* 7:41188. 10.1038/srep41188 PMC5259721 28117402

[B55] Searles Nielsen S. Bammler T. K. Gallagher L. G. Farin F. M. Longstreth W. Jr. Franklin G. M. (2013). Genotype and age at Parkinson disease diagnosis. *Int. J. Mol. Epidemiol. Genet.* 4 61–69. 23565323 PMC3612455

[B56] Sherry S. T. Ward M. H. Kholodov M. Baker J. Phan L. Smigielski E. M. (2001). dbSNP: the NCBI database of genetic variation. *Nucleic Acids Res.* 29 308–311. 10.1093/nar/29.1.308 11125122 PMC29783

[B57] Shi C. H. Tang B. S. Wang L. Lv Z. Y. Wang J. Luo L. Z. (2011). PLA2G6 gene mutation in autosomal recessive early-onset parkinsonism in a Chinese cohort. *Neurology* 77 75–81. 10.1212/wnl.0b013e318221acd3 21700586

[B58] Siitonen A. Nalls M. A. Hernandez D. Gibbs J. R. Ding J. Ylikotila P. (2017). Genetics of early-onset Parkinson’s disease in Finland: exome sequencing and genome-wide association study. *Neurobiol. Aging* 53 195.e7–195.e10. 10.1016/j.neurobiolaging.2017.01.019 PMC5385296 28256260

[B59] Szklarczyk D. Gable A. L. Lyon D. Junge A. Wyder S. Huerta-Cepas J. (2019). STRING v11: protein-protein association networks with increased coverage, supporting functional discovery in genome-wide experimental datasets. *Nucleic Acids Res.* 47 D607–D613. 30476243 10.1093/nar/gky1131 PMC6323986

[B60] Taccioli C. Tegner J. Maselli V. Gomez-Cabrero D. Altobelli G. Emmett W. (2011). ParkDB: a Parkinson’s disease gene expression database. *Database* 2011:bar007. 10.1093/database/bar007 21593080 PMC3098727

[B61] Taghavi S. Chaouni R. Tafakhori A. Azcona L. J. Firouzabadi S. G. Omrani M. D. (2018). A Clinical and Molecular Genetic Study of 50 Families with Autosomal Recessive Parkinsonism Revealed Known and Novel Gene Mutations. *Mol. Neurobiol.* 55 3477–3489. 28502045 10.1007/s12035-017-0535-1 PMC5683945

[B62] Tan M. M. X. Malek N. Lawton M. A. Hubbard L. Pittman A. M. Joseph T. (2019). Genetic analysis of Mendelian mutations in a large UK population-based Parkinson’s disease study. *Brain* 142 2828–2844. 10.1093/brain/awz191 31324919 PMC6735928

[B63] Tang B. Xiong H. Sun P. Zhang Y. Wang D. Hu Z. (2006). Association of PINK1 and DJ-1 confers digenic inheritance of early-onset Parkinson’s disease. *Hum. Mol. Genet.* 15 1816–1825. 10.1093/hmg/ddl104 16632486

[B64] Tang S. Zhang Z. Kavitha G. Tan E. K. Ng S. K. (2009). MDPD: an integrated genetic information resource for Parkinson’s disease. *Nucleic Acids Res.* 37 D858–D862. 18948286 10.1093/nar/gkn770 PMC2686576

[B65] Tian J. Y. Guo J. F. Wang L. Sun Q. Y. Yao L. Y. Luo L. Z. (2012). Mutation analysis of LRRK2, SCNA, UCHL1, HtrA2 and GIGYF2 genes in Chinese patients with autosomal dorminant Parkinson’s disease. *Neurosci. Lett.* 516 207–211. 10.1016/j.neulet.2012.03.086 22503729

[B66] Trinh J. Lohmann K. Baumann H. Balck A. Borsche M. Bruggemann N. (2019). Utility and implications of exome sequencing in early-onset Parkinson’s disease. *Mov. Disord.* 34 133–137. 30537300 10.1002/mds.27559 PMC8950081

[B67] Trinh J. Zeldenrust F. M. J. Huang J. Kasten M. Schaake S. Petkovic S. (2018). Genotype-phenotype relations for the Parkinson’s disease genes SNCA, LRRK2, VPS35: MDSGene systematic review. *Mov. Disord.* 33 1857–1870. 10.1002/mds.27527 30357936

[B68] Wang K. Li M. Hakonarson H. (2010). ANNOVAR: functional annotation of genetic variants from high-throughput sequencing data. *Nucleic Acids Res.* 38:e164. 10.1093/nar/gkq603 20601685 PMC2938201

[B69] Wickremaratchi M. M. Knipe M. D. Sastry B. S. Morgan E. Jones A. Salmon R. (2011). The motor phenotype of Parkinson’s disease in relation to age at onset. *Mov. Disord.* 26 457–463. 10.1002/mds.23469 21229621

[B70] Wilson G. R. Sim J. C. McLean C. Giannandrea M. Galea C. A. Riseley J. R. (2014). Mutations in RAB39B cause X-linked intellectual disability and early-onset Parkinson disease with alpha-synuclein pathology. *Am. J. Hum. Genet.* 95 729–735. 10.1016/j.ajhg.2014.10.015 25434005 PMC4259921

[B71] Wullner U. Kaut O. deBoni L. Piston D. Schmitt I. (2016). DNA methylation in Parkinson’s disease. *J. Neurochem.* 139 108–120. 27120258 10.1111/jnc.13646

[B72] Xu Q. Li K. Sun Q. Ding D. Zhao Y. Yang N. (2017). GCH1 heterozygous variants contributing to Parkinson’s disease. *Brain* 140:e41. 10.1093/brain/awx110 28582483

[B73] Yan W. Tang B. Zhou X. Lei L. Li K. Sun Q. (2017). TMEM230 mutation analysis in Parkinson’s disease in a Chinese population. *Neurobiol. Aging* 49 219.e1–219.e3. 10.1016/j.neurobiolaging.2016.10.007 27814995

[B74] Yang J. O. Kim W. Y. Jeong S. Y. Oh J. H. Jho S. Bhak J. (2009). PDbase: a database of Parkinson’s disease-related genes and genetic variation using substantia nigra ESTs. *BMC Genomics* 10:S32. 10.1186/1471-2164-10-S3-S32 19958497 PMC2788386

[B75] Yang N. Zhao Y. Liu Z. Zhang R. He Y. Zhou Y. (2019). Systematically analyzing rare variants of autosomal-dominant genes for sporadic Parkinson’s disease in a Chinese cohort. *Neurobiol. Aging* 76 215.e1–215.e7. 10.1016/j.neurobiolaging.2018.11.012 30598256

