# Supplementary material for: Gene4PD: A Comprehensive Genetic Database of Parkinson’s Disease
Source: Front Neurosci. 2021 Apr 26;15:679568. doi: 10.3389/fnins.2021.679568 (PMC8107430; doi:10.3389/fnins.2021.679568)
Supplement: Supplementary file 1 [file Table_1.DOCX]

Supplementary Material

| Sections | Description | Pages |
| --- | --- | --- |
| Tables | Table S1. Summary of collected data in present study. | 2 |
|  | Table S2. Weight scheme of various genetic data for prioritizing candidate genes. | 3 |
|  | Table S3. Data source included in Gene4PD. | 4-14 |
|  | Table S4. Gene locus and disease-causing genes of Parkinson disease. | 15 |
|  | Table S5. The biological progress information of gene ontology (GO) terms involved into the genes in PPI network. | 16-50 |
| Figures | Figure S1. The interconnectivities among PD-associated genes accessed by permutation tests. | 51 |
|  | Figure S2. Snapshot of gene-level implications in Gene4PD. | 52 |
|  | Figure S3. Snapshot of analysis panel in Gene4PD. | 53 |

**Supplementary Table 1. Summary of collected data in present study.**

| **Data type** | **Publication reviewed** | **Publication**  **accessed** | **Variants** | **Gene** | **Tissue** |
| --- | --- | --- | --- | --- | --- |
| **Rare variant** | 2,746 | 310 | 954 | 226 | blood |
| **CNV** | 137 | 94 | 139 | 34 | blood |
| **Common variant** | 291 | 42 | 1,237 | 640 | blood |
| **DEG** | 222 | 8 | - | 2,926 | blood, skin, prefrontal cortex, peripheral blood mononuclear cell |
| **DMG** | 206 | 7 | - | 657 | blood, brain, cerebral cortex tissue, peripheral blood lymphocytes, peripheral blood mononuclear cell, saliva |

CNV, copy number variations; DEG, differentially expressed gene; DMG, differentially methylated gene.

**Supplementary Table 2. Weight scheme of various genetic data for prioritizing candidate genes.**

| **Genetic data** | **Score = 5** | **Score = 3** | **Score = 2** | **Score = 1** |
| --- | --- | --- | --- | --- |
| **Rare variants^a^** | LoF | Dmis | Tmis | Remaining variants |
| **CNVs^a^** | CNVs | - | - | - |
| **Associated SNPs^b^** | - | P≤10E-12 | 10E-12<P≤10E-8 | 10E-8<P≤10E-4 |
| **DEGs^b^** | - | P≤10E-6 | 10E-6<P≤10E-4 | 10E-4<P≤10E-2 |
| **DMGs^b^** | - | P≤10E-6 | 10E-6<P≤10E-4 | 10E-4<P≤10E-2 |

Dmis, deleterious missense; LoF, loss of function; Tmis, tolerate missense

1. Genes in one paper were evaluated by the following formula:

- P_(score)_ =$\sum$ ${log}_{2}^{(1+N)}$*score

N is the number of samples carrying a giving rare variant in one study.

1. Genes were evaluated by taking the score corresponding to the minimum P value in each paper for associated SNPs, differential expression and differential DNA methylation.

The final score for each gene was summate its scores in all the collected papers.

**Supplementary Table 3. Data source included in Gene4PD.**

| **Section** | **Database name** | **Detail in database** | **Website** |
| --- | --- | --- | --- |
| **Allele frequency** | gnomAD | The Genome Aggregation Database (gnomAD), is a coalition of investigators seeking to aggregate and harmonize exome and genome sequencing data from a variety of large-scale sequencing projects, and to make summary data available for the wider scientific community.The data set provided on this website spans 123,136 exomes and 15,496 genomes from unrelated individuals sequenced as part of various disease-specific and population genetic studies. | http://gnomad.broadinstitute.org/ |
|  | ExAC | The Exome Aggregation Consortium (ExAC) is a coalition of investigators seeking to aggregate and harmonize exome sequencing data from a variety of large-scale sequencing projects, and to make summary data available for the wider scientific community.The data set provided on this website spans 60,706 unrelated individuals sequenced as part of various disease-specific and population genetic studies. | <http://exac.broadinstitute.org/> |
|  | 1000Genomes | The 1000 Genomes Project was the first project to sequence the genomes of a large number of people, to provide a comprehensive resource on human genetic variation. In the final phase of the project, data from 2,504 samples was combined to allow highly accurate assignment of the genotypes in each sample at all the variant sites the project discovered and the data was from 26 populations,including African, Ad Mixed American, East Asian,European, South Asian, and so on. | <http://www.1000genomes.org/> |
|  | ESP6500 | The dataset in NHLBI GO Exome Sequencing Project (ESP)is from the NHLBI GO Exome Sequencing Project and its ongoing studies which produced and provided exome variant calls for comparison .The current EVS data release (ESP6500SI-V2) is taken from 6503 samples drawn from multiple ESP cohorts and represents all of the ESP exome variant data. | <http://evs.gs.washington.edu/EVS/> |
|  | Kaviar | Kaviar is a compilation of SNVs, indels, and complex variants observed in humans, designed to facilitate testing for the novelty and frequency of observed variants.Kaviar contains 162 million SNV sites (including 25M not in dbSNP) and incorporates data from 35 projects encompassing 77,781 individuals (13.2K whole genome, 64.6K exome). | <http://db.systemsbiology.net/kaviar/> |
|  | HRC | The Haplotype Reference Consortium (HRC) is used for genotype imputation and phasing in other cohorts, typically genome-wide association studies (GWAS), where genotypes are available from genome-wide SNP microarrays.And it contains haplotypes from individuals with predominantly European ancestry, although the HRC includes the 1000 Genomes Project data.The first release consists of 64,976 haplotypes at 39,235,157 SNPs, all with an estimated minor allele count of greater than 5. | [http://www.haplotype-reference-consortium.org](http://www.haplotype-reference-consortium.org/) |
| **Missense prediction** | SIFT | SIFT predicts whether an amino acid substitution affects protein function. SIFT prediction is based on the degree of conservation of amino acid residues in sequence alignments derived from closely related sequences, collected through PSI-BLAST. SIFT can be applied to naturally occurring nonsynonymous polymorphisms or laboratory-induced missense mutations. | [http://sift.jcvi.org](http://sift.jcvi.org/) |
|  | PolyPhen-2 HDIV | PolyPhen-2 is a tool which predicts possible impact of an amino acid substitution on the structure and function of a human protein using straightforward physical and comparative considerations.HumDiv-trained PolyPhen-2 is used for evaluating rare alleles at loci potentially involved in complex phenotypes, dense mapping of regions identified by genome-wide association studies, and analysis of natural selection from sequence data, where even mildly deleterious alleles must be treated as damaging. | <http://genetics.bwh.harvard.edu/pph2> |
|  | PolyPhen-2 HVAR | PolyPhen-2 is a tool which predicts possible impact of an amino acid substitution on the structure and function of a human protein using straightforward physical and comparative considerations.HumVar-trained PolyPhen-2 can diagnose Mendelian diseases that requires distinguishing mutations with drastic effects from all the remaining human variation, including abundant mildly deleterious alleles. | <http://genetics.bwh.harvard.edu/pph2> |
|  | LRT | A likelihood ratio test (LRT) can accurately identify a subset of deleterious mutations that disrupt highly conserved amino acids within protein-coding sequences, which are likely to be unconditionally deleterious. | <http://www.genetics.wustl.edu/jflab/lrt_query.html> |
|  | MutationTaster | MutationTaster employs a Bayes classifier to eventually predict the disease potential of an alteration. The Bayes classifier is fed with the outcome of all tests and the features of the alterations and calculates probabilities for the alteration to be either a disease mutation or a harmless polymorphism. | [http://www.mutationtaster.org](http://www.mutationtaster.org/) |
|  | MutationAssessor | MutationAssessor predicts the functional impact of amino-acid substitutions in proteins, such as mutations discovered in cancer or missense polymorphisms. The functional impact is assessed based on evolutionary conservation of the affected amino acid in protein homologs. | [http://mutationassessor.org](http://mutationassessor.org/) |
|  | FATHMM | Functional Analysis through Hidden Markov Models(FATHMM) is specifically designed for non-synonymous single nucleotide variants (nsSNVs). | [http://fathmm.biocompute.org.uk](http://fathmm.biocompute.org.uk/) |
|  | PROVEAN | Protein Variation Effect Analyzer (PROVEAN) is a software tool which predicts whether an amino acid substitution or indel has an impact on the biological function of a protein. It is useful for filtering sequence variants to identify nonsynonymous or indel variants that are predicted to be functionally important. | <http://provean.jcvi.org/> |
|  | MetaSVM | MetaSVM is a ensemble scoring method for deleterious missense mutations.It integratea nine deleteriousness prediction scores and maximum minor allele frequency for more accurate and comprehensive evaluation of deleteriousness of missense mutations. | <https://www.ncbi.nlm.nih.gov/pubmed/25552646> |
|  | MetaLR | MetaLR is a ensemble scoring method for deleterious missense mutations. It achieves the highest discriminative power compared to all eighteen existing deleteriousness prediction scores, which demonstrated the value of combining information from multiple orthologous approaches. | <https://www.ncbi.nlm.nih.gov/pubmed/25552646> |
|  | VEST 3.0 | The Variant Effect Scoring Tool (VEST) 3.0 is a machine learning method that predicts the functional significance of missense mutations observed through genome sequencing, allowing mutations to be prioritized in subsequent functional studies, based on the probability that they impair protein activity. | [http://wiki.chasmsoftware.org](http://wiki.chasmsoftware.org/) |
|  | M-CAP | M-CAP is a pathogenicity classifier for rare missense variants in the human genome that is tuned to the high sensitivity required in the clinic. By combining previous pathogenicity scores (including SIFT, Polyphen-2 and CADD) with novel features and a powerful model, they attain the best classifier at all thresholds, reducing a typical exome/genome rare (<1%) missense variant (VUS) list from 300 to 120, while never mistaking 95% of known pathogenic variants as benign. | <http://bejerano.stanford.edu/MCAP> |
|  | CADD | Combined Annotation Dependent Depletion (CADD) is a tool for scoring the deleteriousness of single nucleotide variants as well as insertion/deletions variants in the human genome. It is a framework that integrates multiple annotations into one metric by contrasting variants that survived natural selection with simulated mutations. | <http://cadd.gs.washington.edu/> |
|  | GERP++ | GERP++ is a new tool that uses maximum likelihood evolutionary rate estimation for position-specific scoring and, in contrast to previous bottomup methods, a novel dynamic programming approach to subsequently define constrained elements. GERP++ evaluates a richer set of candidate element breakpoints and ranks them based on statistical significance, eliminating the need for biased heuristic extension techniques. | <http://mendel.stanford.edu/SidowLab/downloads/gerp/index.html> |
|  | DANN | DANN is a deep learning approach for annotating the pathogenicity of whole-genome genetic variants.DANN uses the same feature set and training data as CADD to train a deep neural network (DNN). DNNs can capture non-linear relationships among features and are better suited than SVMs for problems with a large number of samples and features. | <https://cbcl.ics.uci.edu/public_data/DANN/> |
|  | fathmm-MKL | fathmm-MKL is capable of predicting the functional effects of protein missense mutations by combining sequence conservation within hidden Markov models (HMMs), representing the alignment of homologous sequences and conserved protein domains, with "pathogenicity weights", representing the overall tolerance of the protein/domain to mutations. | [http://fathmm.biocompute.org.uk](http://fathmm.biocompute.org.uk/) |
|  | Eigen | Eigen is a spectral approach to the functional annotation of genetic variants in coding and noncoding regions. Eigen makes use of a variety of functional annotations in both coding and noncoding regions (such as made available by the ENCODE and Roadmap Epigenomics projects), and combines them into one single measure of functional importance. | <http://www.columbia.edu/~ii2135/eigen.html> |
|  | GenoCanyon | GenoCanyon is a statistical framework to predict functional non-coding regions in the human genome through integrated analysis of annotation data.Meanwhile,it is a whole-genome functional annotation approach based on unsupervised statistical learning. It integrates genomic conservation measures and biochemical annotation data to predict the functional potential at each nucleotide. | <http://genocanyon.med.yale.edu/> |
|  | fitCons | The fitness consequences of functional annotation(fitCons) integrates functional assays (such as ChIP-Seq) with selective pressure inferred using the INSIGHT method. The result is a score ρ in the range [0.0-1.0] that indicates the fraction of genomic positions evincing a particular pattern (or "fingerprint") of functional assay results, that are under selective pressure. | <http://compgen.cshl.edu/fitCons/> |
|  | PhyloP | PhyloP scores measure evolutionary conservation at individual alignment sites.And the phyloP scores are useful to evaluate signatures of selection at particular nucleotides or classes of nucleotides (e.g., third codon positions, or first positions of miRNA target sites). | <http://compgen.bscb.cornell.edu/phast> |
|  | PhastCons | PHAST is a freely available software package for comparative and evolutionary genomics. It consists of about half a dozen major programs, plus more than a dozen utilities for manipulating sequence alignments, phylogenetic trees, and genomic annotations. | <http://compgen.cshl.edu/phast/> |
|  | SiPhy | SiPhy is a approach that takes advantage of deeply sequenced clades to identify evolutionary selection by uncovering not only signatures of rate-based conservation but also substitution patterns characteristic of sequence undergoing natural selection. | <http://portals.broadinstitute.org/genome_bio/siphy/> |
|  | REVEL | REVEL is a new ensemble method for predicting the pathogenicity of missense variants based on a combination of scores from 13 individual tools: MutPred, FATHMM v2.3, VEST 3.0, Polyphen-2, SIFT, PROVEAN, MutationAssessor, MutationTaster, LRT, GERP++, SiPhy, phyloP, and phastCons. REVEL was trained using recently discovered pathogenic and rare neutral missense variants, excluding those previously used to train its constituent tools | <https://sites.google.com/site/revelgenomics/> |
|  | RefSeqGene | The RefSeq Genes contains known human protein-coding and non-protein-coding genes taken from the NCBI RNA reference sequences collection (RefSeq). | <https://www.ncbi.nlm.nih.gov/refseq/rsg/> |
|  | ReVe | ReVe is a method that combinated of REVEL and VEST3, to predict the pathogenicity of missense variants. ReVe showed the best performance for distinguishing pathogenic variants. | [http://varcards.biols.ac.cn](http://varcards.biols.ac.cn/) |
| **Disease-related** | InterVar | InterVar is a bioinformatics software tool for clinical interpretation of genetic variants by the ACMG/AMP 2015 guideline. The input to InterVar is an annotated file generated from ANNOVAR, while the output of InterVar is the classification of variants into 'Benign', 'Likely benign', 'Uncertain significance', 'Likely pathogenic' and 'Pathogenic', together with detailed evidence code. | [http://wintervar.wglab.org](http://wintervar.wglab.org/) |
|  | COSMIC70 | COSMIC is designed to store and display somatic mutation information and related details and contains information relating to human cancers. There are two types of data in COSMIC: Expert manual curation data and systematic screen data.The information in COSMIC is curated by expert scientists, primarily by scrutinizing large numbers of scientific publications. | <http://cancer.sanger.ac.uk/cosmic> |
|  | ICGC | The International Cancer Genome Consortium (ICGC) generates comprehensive catalogues of genomic abnormalities (somatic mutations, abnormal expression of genes, epigenetic modifications) in tumors from 50 different cancer types and/or subtypes which are of clinical and societal importance across the globe and make the data available to the entire research community as rapidly as possible, and with minimal restrictions, to accelerate research into the causes and control of cancer. | [https://icgc.org](https://icgc.org/) |
|  | nci60 | The NCI-60 Human Tumor Cell Lines Screen has served the global cancer research community for >20 years. The screen was implemented in fully operational form in 1990 and utilizes 60 different human tumor cell lines to identify and characterize novel compounds with growth inhibition or killing of tumor cell lines. It is designed to screen up to 3,000 small molecules (synthetic or purified natural products) per year for potential anticancer activity. The operation of this screen utilizes 60 different human tumor cell lines, representing leukemia, melanoma and cancers of the lung, colon, brain, ovary, breast, prostate, and kidney cancers. | <https://dtp.cancer.gov/discovery_development/nci-60> |
|  | dbSNP | The Single Nucleotide Polymorphism database (dbSNP) is a public-domain archive for a broad collection of simple genetic polymorphisms. | <https://www.ncbi.nlm.nih.gov/snp> |
|  | interPro | InterPro is a resource that provides functional analysis of protein sequences by classifying them into families and predicting the presence of domains and important sites. To classify proteins in this way, InterPro uses predictive models, known as signatures, provided by several different databases (referred to as member databases) that make up the InterPro consortium.It combines signatures from multiple, diverse databases into a single searchable resource, reducing redundancy and helping users interpret their sequence analysis results. | <http://www.ebi.ac.uk/interpro/> |
|  | ClinVar | ClinVar is a freely accessible, public archive of reports of the relationships among human variations and phenotypes, with supporting evidence.The database includes germline and somatic variants of any size, type or genomic location. Interpretations are submitted by clinical testing laboratories, research laboratories, locus-specific databases, OMIM, GeneReviews, UniProt, expert panels and practice guidelines. | <https://www.ncbi.nlm.nih.gov/clinvar> |
|  | OMIM | OMIM (online mendelian inheritance in man) is a comprehensive, authoritative compendium of human genes and genetic phenotypes. It contains information on all known mendelian disorders and over 15,000 genes. And it focuses on the relationship between phenotype and genotype. | [https://omim.org](https://omim.org/) |
|  | denovo-db | denovo-db is a collection of germline de novo variants identified in the human genome. As of July 2016, denovo-db contained 40 different studies and 32,991 de novo variants from 23,098 trios. Database features include basic variant information (chromosome location, change, type); detailed annotation at the transcript and protein levels; severity scores; frequency; validation status; and, most importantly, the phenotype of the individual with the variant. | <http://denovo-db.gs.washington.edu/denovo-db/> |
|  | MGI | MGI is the international database resource for the laboratory mouse, providing integrated genetic, genomic, and biological data to facilitate the study of human health and disease. | [http://www.informatics.jax.org](http://www.informatics.jax.org/) |
|  | HPO | The Human Phenotype Ontology (HPO) provides a standardized vocabulary of phenotypic abnormalities encountered in human disease. Each term in the HPO describes a phenotypic abnormality, such as atrial septal defect. The HPO is currently being developed using the medical literature, Orphanet, DECIPHER, and OMIM. HPO currently contains approximately 11,000 terms (still growing) and over 115,000 annotations to hereditary diseases. The HPO also provides a large set of HPO annotations to approximately 4000 common diseases. | <http://human-phenotype-ontology.github.io/> |
| **Basic information** | Entrez | The Entrez Global Query Cross-Database Search System is a federated search engine, or web portal that allows users to search many discrete health sciences databases at the National Center for Biotechnology Information (NCBI) website. The NCBI is a part of the National Library of Medicine (NLM), which is itself a department of the National Institutes of Health (NIH), which in turn is a part of the United States Department of Health and Human Services. The name "Entrez" (a greeting meaning "Come in!" in French) was chosen to reflect the spirit of welcoming the public to search the content available from the NLM. | <https://www.ncbi.nlm.nih.gov/Class/MLACourse/Original8Hour/Entrez/> |
|  | MIM | OMIM is a comprehensive, authoritative compendium of human genes and genetic phenotypes that is freely available and updated daily. The full-text, referenced overviews in OMIM contain information on all known mendelian disorders and over 15,000 genes. OMIM focuses on the relationship between phenotype and genotype. It is updated daily, and the entries contain copious links to other genetics resources. | <http://omim.org/entry> |
|  | HGNC | HGNC is responsible for approving unique symbols and names for human loci, including protein coding genes, ncRNA genes and pseudogenes, to allow unambiguous scientific communication. | [http://www.genenames.org](http://www.genenames.org/) |
|  | Ensembl | Ensembl is a genome browser for vertebrate genomes that supports research in comparative genomics, evolution, sequence variation and transcriptional regulation. Ensembl annotate genes, computes multiple alignments, predicts regulatory function and collects disease data. Ensembl tools include BLAST, BLAT, BioMart and the Variant Effect Predictor (VEP) for all supported species. | [http://asia.ensembl.org](http://asia.ensembl.org/) |
|  | GeneCards | GeneCards is a searchable, integrative database that provides comprehensive, user-friendly information on all annotated and predicted human genes. It automatically integrates gene-centric data from ~125 web sources, including genomic, transcriptomic, proteomic, genetic, clinical and functional information. | <http://www.genecards.org/> |
|  | RVIS | Residual Variation Intolerance Score (RVIS) is a gene-based score intended to help in the interpretation of human sequence data. The intolerance score in its current form is based upon allele frequency data as represented in whole exome sequence data from the NHLBI-ESP6500 data set. The score is designed to rank genes in terms of whether they have more or less common functional genetic variation relative to the genome wide expectation given the amount of apparently neutral variation the gene has. A gene with a positive score has more common functional variation, and a gene with a negative score has less and is referred to as "intolerant". By convention they rank all genes in order from most intolerant to least. | [http://genic-intolerance.org](http://genic-intolerance.org/) |
|  | LoFtool | LoFtool is a novel gene intolerance ranking system which may help in ranking genes of interest based on their LoF intolerance and tissue expression.It predicts genome-wide de novo haploinsufficient mutations accurately and could be of help in search for genetic causes of rare Mendelian diseases. Moreover, its brain expression enrichment coupled to a ROC AUC of 0.86 in detecting neurodevelopmental disorder genes makes LoFtool also an attractive method for investigating complex brain diseases with strong genetic effects. | <https://www.ncbi.nlm.nih.gov/pubmed/27563026> |
|  | heptanucleotide context intolerance score | Varun Aggarwala and Benjamin F Voight applied 7-mer coding substitution probabilities to develop an intolerance score quantifying the difference between the expected and observed numbers of functional variants at a gene, with higher scores consistent with functional constraint. | <https://www.ncbi.nlm.nih.gov/pmc/articles/PMC4811712> |
|  | GDI | the gene damage index (GDI) is describing the accumulated mutational damage for each human gene in the general population, and shows that highly mutated/damaged genes are unlikely to be disease-causing and yet they generate a big proportion of false positive variants harbored in such genes. Therefore removing high GDI genes is a very effective way to remove confidently false positives from WES/WGS data. | <http://lab.rockefeller.edu/casanova/GDI> |
|  | Episcore | EpiScore, a methylation intensity algorithm was developed in methylation-positive men, using area under the curve of the receiver operating characteristic as metric for performance. | <https://omictools.com/episcore-tool#maintainer> |
|  | pLI score | Many of you perform trio exome sequencing to detect de novo mutations in an affected individual. This is for a good reason, as we all know that the probability of a coding DNM to be disease-causing is very high. However, sometimes there are more than a single loss-of-function DNM in an exome, or the gene in which it occurs is simple not related to any disease yet. We looked over the shoulder of experts that are facing such a case. They usually go through the ExAc cohort data and count how many LoF variants they can find in such a gene. Now, there is even a much more elegant way to do so. The ExAc consortium computed a score, called pLI, that indicates the probability that a gene is intolarent to a loss of function mutation. | <http://blog.gene-talk.de/?p=639> |
|  | NCBI Gene | Gene integrates information from a wide range of species. A record may include nomenclature, Reference Sequences (RefSeqs), maps, pathways, variations, phenotypes, and links to genome-, phenotype-, and locus-specific resources worldwide. | <https://www.ncbi.nlm.nih.gov/gene/> |
| **Gene function** | UniProtKB | The UniProt Knowledgebase (UniProtKB) is the central hub for the collection of functional information on proteins, with accurate, consistent and rich annotation. In addition to capturing the core data mandatory for each UniProtKB entry (mainly, the amino acid sequence, protein name or description, taxonomic data and citation information), as much annotation information as possible is added. This includes widely accepted biological ontologies, classifications and cross-references, and clear indications of the quality of annotation in the form of evidence attribution of experimental and computational data. | <http://www.uniprot.org/uniprot/> |
|  | Gene Ontology | The Gene Ontology (GO) project is a major bioinformatics initiative to develop a computational representation of our evolving knowledge of how genes encode biological functions at the molecular, cellular and tissue system levels. The project has developed formal ontologies that represent over 40,000 biological concepts, and are constantly being revised to reflect new discoveries. To date, these concepts have been used to "annotate" gene functions based on experiments reported in over 100,000 peer-reviewed scientific papers. | [http://geneontology.org](http://geneontology.org/) |
|  | InBio Map™ | InBio Map is a high coverage, high quality, convenient and transparent platform for investigating and visualizing protein-protein interactions. | <https://www.intomics.com/inbio/map/#home> |
|  | NCBI BioSystems | The NCBI BioSystems Database provides integrated access to biological systems and their component genes, proteins, and small molecules, as well as literature describing those biosystems and other related data throughout Entrez. | <https://www.ncbi.nlm.nih.gov/biosystems> |
| **Gene expression** | GTEx | The Genotype-Tissue Expression (GTEx) project provides to the scientific community a resource with which to study human gene expression and regulation and its relationship to genetic variation. This project will collect and analyze multiple human tissues from donors who are also densely genotyped, to assess genetic variation within their genomes. By analyzing global RNA expression within individual tissues and treating the expression levels of genes as quantitative traits, variations in gene expression that are highly correlated with genetic variation can be identified as expression quantitative trait loci, or eQTLs. | <http://www.gtexportal.org/> |
|  | The Human Protein Atlas | The Human Protein Atlas contains information for a large majority of all human protein-coding genes regarding the expression and localization of the corresponding proteins based on both RNA and protein data. The atlas consists of three subparts; cell, normal tissue, and cancer with each subpart containing images and data based on antibody-based proteomics and transcriptomics. The tissue atlas contains information of 44 different human tissues and organs with annotation data for altogether 76 different cell types. | <http://www.proteinatlas.org/> |
| **Homology** | HomoloGene | HomoloGene is an automated system for constructing putative homology groups from the complete gene sets of a wide range of eukaryotic species. | <https://www.ncbi.nlm.nih.gov/homologene> |
|  | TreeFam | TreeFam (Tree families database) is a database of phylogenetic trees of animal genes. It aims at developing a curated resource that gives reliable information about ortholog and paralog assignments, and evolutionary history of various gene families. | <http://www.treefam.org/family> |
| **Target drug** | DGIdb | The Drug-Gene Interaction database (DGIdb) mines existing resources that generate hypotheses about how mutated genes might be targeted therapeutically or prioritized for drug development. It provides an interface for searching lists of genes against a compendium of drug-gene interactions and potentially 'druggable' genes.It integrates data from 13 primary sources that cover disease-relevant human genes, drugs, drug-gene interactions and potential druggability. Currently, DGIdb contains over 14,144 drug-gene interactions involving 2,611 genes and 6,307 drugs, and in addition it includes 6,761 genes belonging to one or more of 39 potentially druggable gene categories. A total of 7,668 unique genes have either known or potential druggability. | <http://dgidb.genome.wustl.edu/> |

**Supplementary Table 4. Gene locus and disease-causing genes of Parkinson disease.**

| **Location** | **Full Gene Name Approved by HGNC** | **HGNC Approved Gene Symbol (OMIM)** | **Inheritance** |
| --- | --- | --- | --- |
| 4q22.1 | synuclein alpha | *SNCA* (163890) | AD |
| 6q26 | parkin RBR E3 ubiquitin protein ligase | *PRKN* (602544) | AR |
| 4p13 | ubiquitin C-terminal hydrolase L1 | *UCHL1* (191342) | AD |
| 1p36.23 | parkinsonism associated deglycase | *PARK7* (602533) | AR |
| 12q12 | leucine rich repeat kinase 2 | *LRRK2* (609007) | AD |
| 1p36 | PTEN induced putative kinase 1 | *PINK1* (608309) | AR |
| 15q26.1 | DNA Polymerase Gamma, Catalytic Subunit | *POLG*(174763) | AD |
| 2p13.1 | HtrA serine peptidase 2 | *HTRA2* (606441) | AD |
| 1p36.13 | ATPase 13A2 | *ATP13A2* (610513) | AR |
| 22q12.3 | F-box protein 7 | *FBXO7* (605648) | AR |
| 2q37.1 | GRB10 interacting GYF protein 2 | *GIGYF2* (612003) | AD |
| 1q22 | Glucosylceramidase Beta | *GBA*(606463) | AD |
| 22q13.1 | phospholipase A2 group VI | *PLA2G6* (603604) | AR |
| 3q27.1 | eukaryotic translation initiation factor 4 gamma 1 | *EIF4G1* (600495) | AD |
| 16q11.2 | VPS35, retromer complex component | *VPS35* (601501) | AD |
| 1p31.3 | DnaJ heat shock protein family (Hsp40) member C6 | *DNAJC6* (608375) | AR |
| 21q22.1 | synaptojanin 1 | *SYNJ1* (604297) | AR |
| 3q22.1 | DnaJ Heat Shock Protein Family (Hsp40) Member C13 | *DNAJC13* (614334) | AD |
| 20p13 | transmembrane protein 230 | *TMEM230* (617019) | AD |
| 15q22.2 | vacuolar protein sorting 13 homolog C | *VPS13C* (608879) | AR |
| 14q11.2 | LDL Receptor Related Protein 10 | *LRP10* (609921) | AD |

HGNC: HUGO Gene Nomenclature Committee, AD: autosomal dominant, AR: autosomal recessive

**Supplementary Table 5. The biological progress information of gene ontology (GO) terms involved into the genes in PPI network.**

| **GO biological process complete** | **Homo sapiens (REF) gene number** | **gene number** | **expected** | **over/ under** | **Fold Enrichment** | **raw  P value** | **FDR** |
| --- | --- | --- | --- | --- | --- | --- | --- |
| regulation of neuron death (GO:1901214) | 323 | 17 | 1.41 | + | 12.06 | 9.83E-14 | 7.81E-10 |
| negative regulation of neuron death (GO:1901215) | 215 | 15 | 0.94 | + | 15.99 | 6.76E-14 | 1.07E-09 |
| regulation of autophagy (GO:0010506) | 350 | 16 | 1.53 | + | 10.47 | 4.33E-12 | 2.30E-08 |
| behavior (GO:0007610) | 586 | 19 | 2.56 | + | 7.43 | 1.12E-11 | 4.46E-08 |
| regulation of cellular catabolic process (GO:0031329) | 878 | 22 | 3.83 | + | 5.74 | 2.78E-11 | 8.84E-08 |
| regulation of autophagy of mitochondrion (GO:1903146) | 43 | 8 | 0.19 | + | 42.63 | 5.07E-11 | 1.34E-07 |
| regulation of mitochondrion organization (GO:0010821) | 194 | 12 | 0.85 | + | 14.17 | 9.51E-11 | 2.16E-07 |
| locomotory behavior (GO:0007626) | 200 | 12 | 0.87 | + | 13.75 | 1.33E-10 | 2.64E-07 |
| negative regulation of phosphorylation (GO:0042326) | 452 | 16 | 1.97 | + | 8.11 | 1.68E-10 | 2.97E-07 |
| regulation of biological quality (GO:0065008) | 4102 | 45 | 17.9 | + | 2.51 | 2.14E-10 | 3.40E-07 |
| cellular homeostasis (GO:0019725) | 909 | 21 | 3.97 | + | 5.29 | 3.64E-10 | 5.26E-07 |
| negative regulation of protein phosphorylation (GO:0001933) | 412 | 15 | 1.8 | + | 8.34 | 4.57E-10 | 6.06E-07 |
| catabolic process (GO:0009056) | 2096 | 31 | 9.15 | + | 3.39 | 5.80E-10 | 7.10E-07 |
| cellular chemical homeostasis (GO:0055082) | 752 | 19 | 3.28 | + | 5.79 | 6.85E-10 | 7.26E-07 |
| regulation of catabolic process (GO:0009894) | 1042 | 22 | 4.55 | + | 4.84 | 6.72E-10 | 7.63E-07 |
| synapse organization (GO:0050808) | 294 | 13 | 1.28 | + | 10.13 | 7.72E-10 | 7.67E-07 |
| regulation of reactive oxygen species metabolic process (GO:2000377) | 187 | 11 | 0.82 | + | 13.48 | 1.00E-09 | 9.35E-07 |
| negative regulation of kinase activity (GO:0033673) | 255 | 12 | 1.11 | + | 10.78 | 1.86E-09 | 1.64E-06 |
| transport (GO:0006810) | 4561 | 46 | 19.91 | + | 2.31 | 2.15E-09 | 1.80E-06 |
| chemical homeostasis (GO:0048878) | 1128 | 22 | 4.92 | + | 4.47 | 2.84E-09 | 2.26E-06 |
| negative regulation of phosphorus metabolic process (GO:0010563) | 572 | 16 | 2.5 | + | 6.41 | 4.52E-09 | 3.27E-06 |
| regulation of cellular response to oxidative stress (GO:1900407) | 82 | 8 | 0.36 | + | 22.35 | 5.38E-09 | 3.29E-06 |
| anterograde trans-synaptic signaling (GO:0098916) | 417 | 14 | 1.82 | + | 7.69 | 5.01E-09 | 3.32E-06 |
| negative regulation of phosphate metabolic process (GO:0045936) | 571 | 16 | 2.49 | + | 6.42 | 4.41E-09 | 3.34E-06 |
| negative regulation of cellular protein metabolic process (GO:0032269) | 1060 | 21 | 4.63 | + | 4.54 | 5.36E-09 | 3.41E-06 |
| chemical synaptic transmission (GO:0007268) | 417 | 14 | 1.82 | + | 7.69 | 5.01E-09 | 3.46E-06 |
| establishment of localization (GO:0051234) | 4693 | 46 | 20.48 | + | 2.25 | 7.10E-09 | 4.18E-06 |
| trans-synaptic signaling (GO:0099537) | 436 | 14 | 1.9 | + | 7.36 | 8.65E-09 | 4.74E-06 |
| negative regulation of transferase activity (GO:0051348) | 294 | 12 | 1.28 | + | 9.35 | 8.61E-09 | 4.89E-06 |
| dopamine metabolic process (GO:0042417) | 30 | 6 | 0.13 | + | 45.83 | 9.99E-09 | 5.29E-06 |
| regulation of response to oxidative stress (GO:1902882) | 91 | 8 | 0.4 | + | 20.14 | 1.15E-08 | 5.55E-06 |
| dopamine transport (GO:0015872) | 13 | 5 | 0.06 | + | 88.13 | 1.14E-08 | 5.64E-06 |
| cation homeostasis (GO:0055080) | 705 | 17 | 3.08 | + | 5.53 | 1.21E-08 | 5.65E-06 |
| positive regulation of cellular component organization (GO:0051130) | 1225 | 22 | 5.35 | + | 4.12 | 1.24E-08 | 5.65E-06 |
| negative regulation of protein modification process (GO:0031400) | 611 | 16 | 2.67 | + | 6 | 1.12E-08 | 5.74E-06 |
| negative regulation of protein metabolic process (GO:0051248) | 1129 | 21 | 4.93 | + | 4.26 | 1.58E-08 | 6.79E-06 |
| inorganic ion homeostasis (GO:0098771) | 717 | 17 | 3.13 | + | 5.43 | 1.54E-08 | 6.79E-06 |
| response to oxygen-containing compound (GO:1901700) | 1605 | 25 | 7 | + | 3.57 | 1.66E-08 | 6.93E-06 |
| cellular localization (GO:0051641) | 3015 | 35 | 13.16 | + | 2.66 | 1.76E-08 | 7.16E-06 |
| synaptic signaling (GO:0099536) | 465 | 14 | 2.03 | + | 6.9 | 1.90E-08 | 7.55E-06 |
| cellular cation homeostasis (GO:0030003) | 637 | 16 | 2.78 | + | 5.76 | 1.98E-08 | 7.66E-06 |
| positive regulation of cellular catabolic process (GO:0031331) | 395 | 13 | 1.72 | + | 7.54 | 2.33E-08 | 8.43E-06 |
| regulation of response to reactive oxygen species (GO:1901031) | 35 | 6 | 0.15 | + | 39.28 | 2.27E-08 | 8.58E-06 |
| response to oxidative stress (GO:0006979) | 395 | 13 | 1.72 | + | 7.54 | 2.33E-08 | 8.63E-06 |
| organic hydroxy compound metabolic process (GO:1901615) | 479 | 14 | 2.09 | + | 6.7 | 2.72E-08 | 9.01E-06 |
| catecholamine transport (GO:0051937) | 16 | 5 | 0.07 | + | 71.6 | 2.67E-08 | 9.03E-06 |
| synaptic transmission, dopaminergic (GO:0001963) | 16 | 5 | 0.07 | + | 71.6 | 2.67E-08 | 9.23E-06 |
| cellular ion homeostasis (GO:0006873) | 651 | 16 | 2.84 | + | 5.63 | 2.65E-08 | 9.37E-06 |
| organic hydroxy compound transport (GO:0015850) | 153 | 9 | 0.67 | + | 13.48 | 3.62E-08 | 1.17E-05 |
| dopamine uptake involved in synaptic transmission (GO:0051583) | 5 | 4 | 0.02 | + | > 100 | 4.13E-08 | 1.26E-05 |
| catecholamine uptake involved in synaptic transmission (GO:0051934) | 5 | 4 | 0.02 | + | > 100 | 4.13E-08 | 1.29E-05 |
| homeostatic process (GO:0042592) | 1681 | 25 | 7.34 | + | 3.41 | 4.08E-08 | 1.30E-05 |
| cellular catabolic process (GO:0044248) | 1818 | 26 | 7.93 | + | 3.28 | 4.39E-08 | 1.32E-05 |
| regulation of dopamine metabolic process (GO:0042053) | 19 | 5 | 0.08 | + | 60.3 | 5.52E-08 | 1.54E-05 |
| regulation of catecholamine metabolic process (GO:0042069) | 19 | 5 | 0.08 | + | 60.3 | 5.52E-08 | 1.57E-05 |
| neurotransmitter reuptake (GO:0098810) | 19 | 5 | 0.08 | + | 60.3 | 5.52E-08 | 1.60E-05 |
| vesicle-mediated transport (GO:0016192) | 1972 | 27 | 8.61 | + | 3.14 | 5.44E-08 | 1.60E-05 |
| ion homeostasis (GO:0050801) | 790 | 17 | 3.45 | + | 4.93 | 6.10E-08 | 1.67E-05 |
| regulation of protein phosphorylation (GO:0001932) | 1467 | 23 | 6.4 | + | 3.59 | 6.42E-08 | 1.70E-05 |
| neuromuscular process (GO:0050905) | 115 | 8 | 0.5 | + | 15.94 | 6.36E-08 | 1.72E-05 |
| positive regulation of autophagy of mitochondrion (GO:1903599) | 20 | 5 | 0.09 | + | 57.28 | 6.88E-08 | 1.79E-05 |
| positive regulation of mitochondrion organization (GO:0010822) | 119 | 8 | 0.52 | + | 15.4 | 8.17E-08 | 2.09E-05 |
| regulation of synapse organization (GO:0050807) | 229 | 10 | 1 | + | 10.01 | 8.89E-08 | 2.17E-05 |
| catechol-containing compound metabolic process (GO:0009712) | 45 | 6 | 0.2 | + | 30.55 | 8.77E-08 | 2.18E-05 |
| catecholamine metabolic process (GO:0006584) | 45 | 6 | 0.2 | + | 30.55 | 8.77E-08 | 2.21E-05 |
| metal ion homeostasis (GO:0055065) | 622 | 15 | 2.71 | + | 5.53 | 9.68E-08 | 2.33E-05 |
| process utilizing autophagic mechanism (GO:0061919) | 302 | 11 | 1.32 | + | 8.35 | 1.14E-07 | 2.45E-05 |
| monoamine transport (GO:0015844) | 22 | 5 | 0.1 | + | 52.08 | 1.04E-07 | 2.46E-05 |
| negative regulation of protein kinase activity (GO:0006469) | 235 | 10 | 1.03 | + | 9.75 | 1.12E-07 | 2.47E-05 |
| autophagy (GO:0006914) | 302 | 11 | 1.32 | + | 8.35 | 1.14E-07 | 2.48E-05 |
| localization (GO:0051179) | 5850 | 50 | 25.53 | + | 1.96 | 1.11E-07 | 2.49E-05 |
| regulation of macroautophagy (GO:0016241) | 177 | 9 | 0.77 | + | 11.65 | 1.19E-07 | 2.52E-05 |
| negative regulation of cellular response to oxidative stress (GO:1900408) | 47 | 6 | 0.21 | + | 29.25 | 1.11E-07 | 2.52E-05 |
| regulation of phosphorylation (GO:0042325) | 1638 | 24 | 7.15 | + | 3.36 | 1.08E-07 | 2.53E-05 |
| negative regulation of oxidative stress-induced cell death (GO:1903202) | 47 | 6 | 0.21 | + | 29.25 | 1.11E-07 | 2.56E-05 |
| memory (GO:0007613) | 126 | 8 | 0.55 | + | 14.55 | 1.24E-07 | 2.59E-05 |
| mitochondrion organization (GO:0007005) | 460 | 13 | 2.01 | + | 6.48 | 1.30E-07 | 2.69E-05 |
| regulation of synapse structure or activity (GO:0050803) | 240 | 10 | 1.05 | + | 9.55 | 1.35E-07 | 2.76E-05 |
| regulation of neurotransmitter levels (GO:0001505) | 242 | 10 | 1.06 | + | 9.47 | 1.46E-07 | 2.86E-05 |
| positive regulation of organelle organization (GO:0010638) | 642 | 15 | 2.8 | + | 5.35 | 1.44E-07 | 2.87E-05 |
| positive regulation of catabolic process (GO:0009896) | 464 | 13 | 2.03 | + | 6.42 | 1.44E-07 | 2.89E-05 |
| cellular response to oxidative stress (GO:0034599) | 243 | 10 | 1.06 | + | 9.43 | 1.51E-07 | 2.93E-05 |
| negative regulation of response to oxidative stress (GO:1902883) | 50 | 6 | 0.22 | + | 27.5 | 1.55E-07 | 2.98E-05 |
| cellular metal ion homeostasis (GO:0006875) | 558 | 14 | 2.44 | + | 5.75 | 1.70E-07 | 3.21E-05 |
| regulation of cellular response to stress (GO:0080135) | 752 | 16 | 3.28 | + | 4.88 | 1.83E-07 | 3.42E-05 |
| regulation of cellular protein metabolic process (GO:0032268) | 2685 | 31 | 11.72 | + | 2.65 | 1.92E-07 | 3.50E-05 |
| establishment of localization in cell (GO:0051649) | 2385 | 29 | 10.41 | + | 2.79 | 1.90E-07 | 3.51E-05 |
| vesicle-mediated transport in synapse (GO:0099003) | 136 | 8 | 0.59 | + | 13.48 | 2.16E-07 | 3.90E-05 |
| regulation of mitochondrial fission (GO:0090140) | 27 | 5 | 0.12 | + | 42.43 | 2.54E-07 | 4.55E-05 |
| regulation of cellular protein catabolic process (GO:1903362) | 261 | 10 | 1.14 | + | 8.78 | 2.85E-07 | 4.99E-05 |
| negative regulation of cellular catabolic process (GO:0031330) | 261 | 10 | 1.14 | + | 8.78 | 2.85E-07 | 5.04E-05 |
| neurotransmitter uptake (GO:0001504) | 28 | 5 | 0.12 | + | 40.92 | 2.99E-07 | 5.17E-05 |
| dopamine biosynthetic process (GO:0042416) | 10 | 4 | 0.04 | + | 91.65 | 3.23E-07 | 5.35E-05 |
| catecholamine uptake (GO:0090493) | 10 | 4 | 0.04 | + | 91.65 | 3.23E-07 | 5.41E-05 |
| dopamine uptake (GO:0090494) | 10 | 4 | 0.04 | + | 91.65 | 3.23E-07 | 5.46E-05 |
| positive regulation of cellular amine metabolic process (GO:0033240) | 10 | 4 | 0.04 | + | 91.65 | 3.23E-07 | 5.52E-05 |
| learning or memory (GO:0007611) | 266 | 10 | 1.16 | + | 8.61 | 3.38E-07 | 5.53E-05 |
| membrane lipid metabolic process (GO:0006643) | 204 | 9 | 0.89 | + | 10.11 | 3.75E-07 | 6.08E-05 |
| cellular response to stress (GO:0033554) | 1756 | 24 | 7.66 | + | 3.13 | 3.85E-07 | 6.18E-05 |
| cell junction organization (GO:0034330) | 510 | 13 | 2.23 | + | 5.84 | 4.11E-07 | 6.53E-05 |
| neurotransmitter transport (GO:0006836) | 149 | 8 | 0.65 | + | 12.3 | 4.19E-07 | 6.59E-05 |
| regulation of cellular localization (GO:0060341) | 1025 | 18 | 4.47 | + | 4.02 | 4.61E-07 | 7.18E-05 |
| positive regulation of cell death (GO:0010942) | 715 | 15 | 3.12 | + | 4.81 | 5.53E-07 | 8.53E-05 |
| cellular response to manganese ion (GO:0071287) | 12 | 4 | 0.05 | + | 76.38 | 5.83E-07 | 8.92E-05 |
| regulation of postsynapse organization (GO:0099175) | 107 | 7 | 0.47 | + | 14.99 | 6.53E-07 | 9.89E-05 |
| regulation of protein metabolic process (GO:0051246) | 2842 | 31 | 12.4 | + | 2.5 | 6.74E-07 | 1.01E-04 |
| cellular response to chemical stress (GO:0062197) | 289 | 10 | 1.26 | + | 7.93 | 7.02E-07 | 1.04E-04 |
| positive regulation of proteolysis (GO:0045862) | 367 | 11 | 1.6 | + | 6.87 | 7.45E-07 | 1.10E-04 |
| regulation of oxidative stress-induced cell death (GO:1903201) | 68 | 6 | 0.3 | + | 20.22 | 8.32E-07 | 1.21E-04 |
| regulation of phosphate metabolic process (GO:0019220) | 1837 | 24 | 8.02 | + | 2.99 | 8.62E-07 | 1.25E-04 |
| regulation of phosphorus metabolic process (GO:0051174) | 1838 | 24 | 8.02 | + | 2.99 | 8.70E-07 | 1.25E-04 |
| organonitrogen compound metabolic process (GO:1901564) | 5444 | 46 | 23.76 | + | 1.94 | 8.83E-07 | 1.25E-04 |
| regulation of superoxide metabolic process (GO:0090322) | 36 | 5 | 0.16 | + | 31.82 | 9.18E-07 | 1.28E-04 |
| regulation of proteolysis (GO:0030162) | 745 | 15 | 3.25 | + | 4.61 | 9.16E-07 | 1.29E-04 |
| regulation of autophagy of mitochondrion in response to mitochondrial depolarization (GO:1904923) | 14 | 4 | 0.06 | + | 65.47 | 9.74E-07 | 1.34E-04 |
| regulation of cell death (GO:0010941) | 1714 | 23 | 7.48 | + | 3.07 | 9.85E-07 | 1.34E-04 |
| regulation of protein stability (GO:0031647) | 300 | 10 | 1.31 | + | 7.64 | 9.74E-07 | 1.35E-04 |
| regulation of cellular component organization (GO:0051128) | 2440 | 28 | 10.65 | + | 2.63 | 1.08E-06 | 1.45E-04 |
| lipid metabolic process (GO:0006629) | 1219 | 19 | 5.32 | + | 3.57 | 1.24E-06 | 1.65E-04 |
| cognition (GO:0050890) | 309 | 10 | 1.35 | + | 7.42 | 1.26E-06 | 1.67E-04 |
| regulation of protein modification process (GO:0031399) | 1885 | 24 | 8.23 | + | 2.92 | 1.36E-06 | 1.79E-04 |
| mitochondrion to lysosome transport (GO:0099074) | 3 | 3 | 0.01 | + | > 100 | 1.57E-06 | 2.03E-04 |
| mitochondrion-derived vesicle mediated transport (GO:0099075) | 3 | 3 | 0.01 | + | > 100 | 1.57E-06 | 2.05E-04 |
| divalent inorganic cation homeostasis (GO:0072507) | 485 | 12 | 2.12 | + | 5.67 | 1.61E-06 | 2.07E-04 |
| negative regulation of catabolic process (GO:0009895) | 320 | 10 | 1.4 | + | 7.16 | 1.71E-06 | 2.18E-04 |
| regulation of response to stress (GO:0080134) | 1636 | 22 | 7.14 | + | 3.08 | 1.74E-06 | 2.20E-04 |
| organic hydroxy compound catabolic process (GO:1901616) | 78 | 6 | 0.34 | + | 17.63 | 1.76E-06 | 2.21E-04 |
| synaptic vesicle cycle (GO:0099504) | 126 | 7 | 0.55 | + | 12.73 | 1.85E-06 | 2.29E-04 |
| regulation of protein catabolic process (GO:0042176) | 405 | 11 | 1.77 | + | 6.22 | 1.90E-06 | 2.30E-04 |
| negative regulation of response to reactive oxygen species (GO:1901032) | 17 | 4 | 0.07 | + | 53.91 | 1.89E-06 | 2.31E-04 |
| negative regulation of hydrogen peroxide-induced cell death (GO:1903206) | 17 | 4 | 0.07 | + | 53.91 | 1.89E-06 | 2.33E-04 |
| adult locomotory behavior (GO:0008344) | 80 | 6 | 0.35 | + | 17.18 | 2.03E-06 | 2.44E-04 |
| cell-cell signaling (GO:0007267) | 1143 | 18 | 4.99 | + | 3.61 | 2.15E-06 | 2.57E-04 |
| regulation of establishment of protein localization (GO:0070201) | 695 | 14 | 3.03 | + | 4.62 | 2.19E-06 | 2.60E-04 |
| regulation of mitophagy (GO:1901524) | 18 | 4 | 0.08 | + | 50.92 | 2.30E-06 | 2.71E-04 |
| regulation of kinase activity (GO:0043549) | 916 | 16 | 4 | + | 4 | 2.36E-06 | 2.76E-04 |
| regulation of peptide transport (GO:0090087) | 703 | 14 | 3.07 | + | 4.56 | 2.50E-06 | 2.90E-04 |
| membrane lipid biosynthetic process (GO:0046467) | 133 | 7 | 0.58 | + | 12.06 | 2.60E-06 | 3.00E-04 |
| catecholamine biosynthetic process (GO:0042423) | 19 | 4 | 0.08 | + | 48.24 | 2.77E-06 | 3.08E-04 |
| catechol-containing compound biosynthetic process (GO:0009713) | 19 | 4 | 0.08 | + | 48.24 | 2.77E-06 | 3.10E-04 |
| negative regulation of oxidative stress-induced intrinsic apoptotic signaling pathway (GO:1902176) | 19 | 4 | 0.08 | + | 48.24 | 2.77E-06 | 3.13E-04 |
| positive regulation of cellular amino acid metabolic process (GO:0045764) | 4 | 3 | 0.02 | + | > 100 | 2.74E-06 | 3.14E-04 |
| response to manganese ion (GO:0010042) | 19 | 4 | 0.08 | + | 48.24 | 2.77E-06 | 3.15E-04 |
| negative regulation of catalytic activity (GO:0043086) | 820 | 15 | 3.58 | + | 4.19 | 2.93E-06 | 3.23E-04 |
| negative regulation of intracellular signal transduction (GO:1902532) | 515 | 12 | 2.25 | + | 5.34 | 2.95E-06 | 3.24E-04 |
| regulation of cellular amine metabolic process (GO:0033238) | 86 | 6 | 0.38 | + | 15.99 | 3.01E-06 | 3.28E-04 |
| negative regulation of response to stimulus (GO:0048585) | 1842 | 23 | 8.04 | + | 2.86 | 3.33E-06 | 3.53E-04 |
| response to mitochondrial depolarisation (GO:0098780) | 20 | 4 | 0.09 | + | 45.83 | 3.32E-06 | 3.54E-04 |
| regulation of neuron projection development (GO:0010975) | 521 | 12 | 2.27 | + | 5.28 | 3.32E-06 | 3.56E-04 |
| regulation of transport (GO:0051049) | 1841 | 23 | 8.03 | + | 2.86 | 3.30E-06 | 3.56E-04 |
| negative regulation of cellular component organization (GO:0051129) | 724 | 14 | 3.16 | + | 4.43 | 3.49E-06 | 3.67E-04 |
| phenol-containing compound metabolic process (GO:0018958) | 89 | 6 | 0.39 | + | 15.45 | 3.63E-06 | 3.77E-04 |
| regulation of intracellular protein transport (GO:0033157) | 271 | 9 | 1.18 | + | 7.61 | 3.61E-06 | 3.78E-04 |
| vacuole organization (GO:0007033) | 142 | 7 | 0.62 | + | 11.3 | 3.94E-06 | 4.04E-04 |
| negative regulation of oxidative stress-induced neuron death (GO:1903204) | 21 | 4 | 0.09 | + | 43.64 | 3.94E-06 | 4.06E-04 |
| cellular response to oxygen-containing compound (GO:1901701) | 1076 | 17 | 4.7 | + | 3.62 | 4.13E-06 | 4.16E-04 |
| regulation of membrane potential (GO:0042391) | 440 | 11 | 1.92 | + | 5.73 | 4.11E-06 | 4.17E-04 |
| adult behavior (GO:0030534) | 143 | 7 | 0.62 | + | 11.22 | 4.11E-06 | 4.19E-04 |
| negative regulation of oxidative stress-induced neuron intrinsic apoptotic signaling pathway (GO:1903377) | 5 | 3 | 0.02 | + | > 100 | 4.38E-06 | 4.29E-04 |
| regulation of mitochondrial electron transport, NADH to ubiquinone (GO:1902956) | 5 | 3 | 0.02 | + | > 100 | 4.38E-06 | 4.32E-04 |
| positive regulation of transport (GO:0051050) | 962 | 16 | 4.2 | + | 3.81 | 4.37E-06 | 4.34E-04 |
| regulation of nervous system development (GO:0051960) | 962 | 16 | 4.2 | + | 3.81 | 4.37E-06 | 4.37E-04 |
| regulation of organelle organization (GO:0033043) | 1336 | 19 | 5.83 | + | 3.26 | 4.66E-06 | 4.54E-04 |
| cell communication (GO:0007154) | 5609 | 45 | 24.48 | + | 1.84 | 4.80E-06 | 4.65E-04 |
| regulation of reactive oxygen species biosynthetic process (GO:1903426) | 94 | 6 | 0.41 | + | 14.63 | 4.89E-06 | 4.71E-04 |
| regulation of intracellular transport (GO:0032386) | 363 | 10 | 1.58 | + | 6.31 | 5.08E-06 | 4.87E-04 |
| regulation of hydrogen peroxide-induced cell death (GO:1903205) | 23 | 4 | 0.1 | + | 39.85 | 5.42E-06 | 5.16E-04 |
| positive regulation of cellular protein catabolic process (GO:1903364) | 150 | 7 | 0.65 | + | 10.69 | 5.56E-06 | 5.26E-04 |
| regulation of cellular component biogenesis (GO:0044087) | 982 | 16 | 4.29 | + | 3.73 | 5.65E-06 | 5.31E-04 |
| modulation of chemical synaptic transmission (GO:0050804) | 456 | 11 | 1.99 | + | 5.53 | 5.73E-06 | 5.36E-04 |
| regulation of neuron apoptotic process (GO:0043523) | 215 | 8 | 0.94 | + | 8.53 | 5.80E-06 | 5.36E-04 |
| regulation of trans-synaptic signaling (GO:0099177) | 457 | 11 | 1.99 | + | 5.52 | 5.85E-06 | 5.37E-04 |
| negative regulation of neuron apoptotic process (GO:0043524) | 151 | 7 | 0.66 | + | 10.62 | 5.80E-06 | 5.39E-04 |
| positive regulation of cellular protein metabolic process (GO:0032270) | 1631 | 21 | 7.12 | + | 2.95 | 6.19E-06 | 5.66E-04 |
| negative regulation of transport (GO:0051051) | 463 | 11 | 2.02 | + | 5.44 | 6.60E-06 | 5.86E-04 |
| response to metal ion (GO:0010038) | 374 | 10 | 1.63 | + | 6.13 | 6.56E-06 | 5.86E-04 |
| positive regulation of histone deacetylase activity (GO:1901727) | 6 | 3 | 0.03 | + | > 100 | 6.54E-06 | 5.88E-04 |
| regulation of hydrogen peroxide-induced neuron death (GO:1903207) | 6 | 3 | 0.03 | + | > 100 | 6.54E-06 | 5.91E-04 |
| organic substance catabolic process (GO:1901575) | 1780 | 22 | 7.77 | + | 2.83 | 6.76E-06 | 5.94E-04 |
| cellular divalent inorganic cation homeostasis (GO:0072503) | 464 | 11 | 2.03 | + | 5.43 | 6.73E-06 | 5.94E-04 |
| negative regulation of hydrogen peroxide-induced neuron death (GO:1903208) | 6 | 3 | 0.03 | + | > 100 | 6.54E-06 | 5.94E-04 |
| sphingolipid metabolic process (GO:0006665) | 155 | 7 | 0.68 | + | 10.35 | 6.83E-06 | 5.97E-04 |
| negative regulation of reactive oxygen species metabolic process (GO:2000378) | 57 | 5 | 0.25 | + | 20.1 | 7.38E-06 | 6.41E-04 |
| regulation of protein transport (GO:0051223) | 670 | 13 | 2.92 | + | 4.45 | 7.79E-06 | 6.73E-04 |
| synapse assembly (GO:0007416) | 103 | 6 | 0.45 | + | 13.35 | 8.06E-06 | 6.92E-04 |
| regulation of catalytic activity (GO:0050790) | 2394 | 26 | 10.45 | + | 2.49 | 8.15E-06 | 6.97E-04 |
| regulation of proteolysis involved in cellular protein catabolic process (GO:1903050) | 226 | 8 | 0.99 | + | 8.11 | 8.25E-06 | 6.98E-04 |
| regulation of cellular protein localization (GO:1903827) | 571 | 12 | 2.49 | + | 4.82 | 8.24E-06 | 7.01E-04 |
| response to organic substance (GO:0010033) | 3027 | 30 | 13.21 | + | 2.27 | 8.51E-06 | 7.16E-04 |
| negative regulation of cell death (GO:0060548) | 1017 | 16 | 4.44 | + | 3.6 | 8.71E-06 | 7.29E-04 |
| regulation of neuron differentiation (GO:0045664) | 678 | 13 | 2.96 | + | 4.39 | 8.82E-06 | 7.34E-04 |
| regulation of synaptic vesicle transport (GO:1902803) | 7 | 3 | 0.03 | + | 98.2 | 9.32E-06 | 7.68E-04 |
| regulation of mitochondrial ATP synthesis coupled electron transport (GO:1905446) | 7 | 3 | 0.03 | + | 98.2 | 9.32E-06 | 7.72E-04 |
| regulation of oxidative stress-induced intrinsic apoptotic signaling pathway (GO:1902175) | 27 | 4 | 0.12 | + | 33.95 | 9.59E-06 | 7.86E-04 |
| negative regulation of mitochondrion organization (GO:0010823) | 61 | 5 | 0.27 | + | 18.78 | 1.01E-05 | 8.20E-04 |
| regulation of transferase activity (GO:0051338) | 1033 | 16 | 4.51 | + | 3.55 | 1.06E-05 | 8.56E-04 |
| nervous system development (GO:0007399) | 2430 | 26 | 10.61 | + | 2.45 | 1.06E-05 | 8.59E-04 |
| regulation of oxidoreductase activity (GO:0051341) | 109 | 6 | 0.48 | + | 12.61 | 1.10E-05 | 8.80E-04 |
| maintenance of location (GO:0051235) | 168 | 7 | 0.73 | + | 9.55 | 1.13E-05 | 9.04E-04 |
| regulation of protein kinase activity (GO:0045859) | 806 | 14 | 3.52 | + | 3.98 | 1.16E-05 | 9.21E-04 |
| negative regulation of MAPK cascade (GO:0043409) | 169 | 7 | 0.74 | + | 9.49 | 1.17E-05 | 9.29E-04 |
| positive regulation of phosphate metabolic process (GO:0045937) | 1168 | 17 | 5.1 | + | 3.33 | 1.20E-05 | 9.34E-04 |
| positive regulation of phosphorus metabolic process (GO:0010562) | 1168 | 17 | 5.1 | + | 3.33 | 1.20E-05 | 9.38E-04 |
| regulation of plasma membrane bounded cell projection organization (GO:0120035) | 698 | 13 | 3.05 | + | 4.27 | 1.20E-05 | 9.41E-04 |
| startle response (GO:0001964) | 29 | 4 | 0.13 | + | 31.6 | 1.24E-05 | 9.57E-04 |
| regulation of oxidative stress-induced neuron death (GO:1903203) | 29 | 4 | 0.13 | + | 31.6 | 1.24E-05 | 9.61E-04 |
| regulation of oxidative stress-induced neuron intrinsic apoptotic signaling pathway (GO:1903376) | 8 | 3 | 0.03 | + | 85.92 | 1.28E-05 | 9.81E-04 |
| regulation of cell projection organization (GO:0031344) | 707 | 13 | 3.09 | + | 4.21 | 1.37E-05 | 1.04E-03 |
| response to toxic substance (GO:0009636) | 244 | 8 | 1.06 | + | 7.51 | 1.41E-05 | 1.07E-03 |
| positive regulation of protein metabolic process (GO:0051247) | 1722 | 21 | 7.52 | + | 2.79 | 1.41E-05 | 1.07E-03 |
| negative regulation of synaptic transmission (GO:0050805) | 66 | 5 | 0.29 | + | 17.36 | 1.44E-05 | 1.08E-03 |
| import into cell (GO:0098657) | 177 | 7 | 0.77 | + | 9.06 | 1.57E-05 | 1.18E-03 |
| regulation of lysosomal protein catabolic process (GO:1905165) | 9 | 3 | 0.04 | + | 76.38 | 1.70E-05 | 1.25E-03 |
| regulation of localization (GO:0032879) | 2796 | 28 | 12.2 | + | 2.29 | 1.68E-05 | 1.25E-03 |
| positive regulation of deacetylase activity (GO:0090045) | 9 | 3 | 0.04 | + | 76.38 | 1.70E-05 | 1.26E-03 |
| regulation of histone deacetylase activity (GO:1901725) | 9 | 3 | 0.04 | + | 76.38 | 1.70E-05 | 1.26E-03 |
| alcohol metabolic process (GO:0006066) | 332 | 9 | 1.45 | + | 6.21 | 1.76E-05 | 1.29E-03 |
| regulation of neurogenesis (GO:0050767) | 847 | 14 | 3.7 | + | 3.79 | 2.00E-05 | 1.46E-03 |
| regulation of protein serine/threonine kinase activity (GO:0071900) | 526 | 11 | 2.3 | + | 4.79 | 2.11E-05 | 1.53E-03 |
| response to stress (GO:0006950) | 3656 | 33 | 15.96 | + | 2.07 | 2.12E-05 | 1.53E-03 |
| cellular component organization or biogenesis (GO:0071840) | 5908 | 45 | 25.78 | + | 1.75 | 2.15E-05 | 1.54E-03 |
| regulation of secretion (GO:0051046) | 739 | 13 | 3.23 | + | 4.03 | 2.16E-05 | 1.54E-03 |
| cellular calcium ion homeostasis (GO:0006874) | 431 | 10 | 1.88 | + | 5.32 | 2.17E-05 | 1.55E-03 |
| neuron projection maintenance (GO:1990535) | 10 | 3 | 0.04 | + | 68.74 | 2.20E-05 | 1.55E-03 |
| phenol-containing compound biosynthetic process (GO:0046189) | 34 | 4 | 0.15 | + | 26.96 | 2.20E-05 | 1.56E-03 |
| cellular lipid metabolic process (GO:0044255) | 975 | 15 | 4.26 | + | 3.53 | 2.23E-05 | 1.57E-03 |
| macroautophagy (GO:0016236) | 189 | 7 | 0.82 | + | 8.49 | 2.35E-05 | 1.65E-03 |
| regulation of establishment of protein localization to mitochondrion (GO:1903747) | 74 | 5 | 0.32 | + | 15.48 | 2.43E-05 | 1.68E-03 |
| regulation of mitochondrial membrane potential (GO:0051881) | 74 | 5 | 0.32 | + | 15.48 | 2.43E-05 | 1.69E-03 |
| regulation of proteasomal protein catabolic process (GO:0061136) | 192 | 7 | 0.84 | + | 8.35 | 2.60E-05 | 1.79E-03 |
| cellular component organization (GO:0016043) | 5688 | 44 | 24.82 | + | 1.77 | 2.59E-05 | 1.79E-03 |
| response to chemical (GO:0042221) | 4415 | 37 | 19.27 | + | 1.92 | 2.71E-05 | 1.86E-03 |
| presynaptic membrane assembly (GO:0097105) | 11 | 3 | 0.05 | + | 62.49 | 2.79E-05 | 1.87E-03 |
| regulation of protein localization (GO:0032880) | 993 | 15 | 4.33 | + | 3.46 | 2.75E-05 | 1.88E-03 |
| regulation of protein catabolic process in the vacuole (GO:1904350) | 11 | 3 | 0.05 | + | 62.49 | 2.79E-05 | 1.88E-03 |
| regulation of removal of superoxide radicals (GO:2000121) | 11 | 3 | 0.05 | + | 62.49 | 2.79E-05 | 1.89E-03 |
| positive regulation of protein modification process (GO:0031401) | 1250 | 17 | 5.46 | + | 3.12 | 2.83E-05 | 1.89E-03 |
| calcium ion homeostasis (GO:0055074) | 444 | 10 | 1.94 | + | 5.16 | 2.78E-05 | 1.89E-03 |
| response to inorganic substance (GO:0010035) | 546 | 11 | 2.38 | + | 4.62 | 2.95E-05 | 1.96E-03 |
| regulation of dendritic spine development (GO:0060998) | 78 | 5 | 0.34 | + | 14.69 | 3.08E-05 | 2.04E-03 |
| regulation of intracellular signal transduction (GO:1902531) | 1823 | 21 | 7.96 | + | 2.64 | 3.28E-05 | 2.15E-03 |
| response to organonitrogen compound (GO:0010243) | 1008 | 15 | 4.4 | + | 3.41 | 3.27E-05 | 2.15E-03 |
| positive regulation of autophagy (GO:0010508) | 134 | 6 | 0.58 | + | 10.26 | 3.35E-05 | 2.19E-03 |
| positive regulation of programmed cell death (GO:0043068) | 661 | 12 | 2.88 | + | 4.16 | 3.41E-05 | 2.22E-03 |
| negative regulation of protein serine/threonine kinase activity (GO:0071901) | 135 | 6 | 0.59 | + | 10.18 | 3.49E-05 | 2.24E-03 |
| presynaptic membrane organization (GO:0097090) | 12 | 3 | 0.05 | + | 57.28 | 3.48E-05 | 2.24E-03 |
| regulation of deacetylase activity (GO:0150065) | 12 | 3 | 0.05 | + | 57.28 | 3.48E-05 | 2.25E-03 |
| regulation of protein-containing complex assembly (GO:0043254) | 456 | 10 | 1.99 | + | 5.02 | 3.47E-05 | 2.25E-03 |
| response to organic cyclic compound (GO:0014070) | 894 | 14 | 3.9 | + | 3.59 | 3.58E-05 | 2.29E-03 |
| nervous system process (GO:0050877) | 1411 | 18 | 6.16 | + | 2.92 | 3.66E-05 | 2.33E-03 |
| regulation of vesicle-mediated transport (GO:0060627) | 561 | 11 | 2.45 | + | 4.49 | 3.76E-05 | 2.38E-03 |
| endocytosis (GO:0006897) | 563 | 11 | 2.46 | + | 4.48 | 3.88E-05 | 2.45E-03 |
| regulation of molecular function (GO:0065009) | 3079 | 29 | 13.44 | + | 2.16 | 3.91E-05 | 2.46E-03 |
| response to stimulus (GO:0050896) | 8545 | 57 | 37.29 | + | 1.53 | 3.93E-05 | 2.46E-03 |
| glial cell activation (GO:0061900) | 40 | 4 | 0.17 | + | 22.91 | 3.96E-05 | 2.46E-03 |
| neuron projection organization (GO:0106027) | 40 | 4 | 0.17 | + | 22.91 | 3.96E-05 | 2.47E-03 |
| metabolic process (GO:0008152) | 8587 | 57 | 37.48 | + | 1.52 | 4.21E-05 | 2.61E-03 |
| positive regulation of autophagy of mitochondrion in response to mitochondrial depolarization (GO:1904925) | 13 | 3 | 0.06 | + | 52.88 | 4.27E-05 | 2.63E-03 |
| negative regulation of autophagy (GO:0010507) | 84 | 5 | 0.37 | + | 13.64 | 4.32E-05 | 2.65E-03 |
| negative regulation of cellular metabolic process (GO:0031324) | 2624 | 26 | 11.45 | + | 2.27 | 4.50E-05 | 2.75E-03 |
| negative regulation of metabolic process (GO:0009892) | 3118 | 29 | 13.61 | + | 2.13 | 4.63E-05 | 2.82E-03 |
| regulation of secretion by cell (GO:1903530) | 683 | 12 | 2.98 | + | 4.03 | 4.65E-05 | 2.82E-03 |
| generation of neurons (GO:0048699) | 1585 | 19 | 6.92 | + | 2.75 | 4.97E-05 | 3.00E-03 |
| cellular response to chemical stimulus (GO:0070887) | 2929 | 28 | 12.78 | + | 2.19 | 5.02E-05 | 3.02E-03 |
| nitrogen compound metabolic process (GO:0006807) | 7089 | 50 | 30.94 | + | 1.62 | 5.16E-05 | 3.05E-03 |
| neuroinflammatory response (GO:0150076) | 43 | 4 | 0.19 | + | 21.31 | 5.16E-05 | 3.06E-03 |
| negative regulation of molecular function (GO:0044092) | 1178 | 16 | 5.14 | + | 3.11 | 5.12E-05 | 3.07E-03 |
| mitochondrion disassembly (GO:0061726) | 43 | 4 | 0.19 | + | 21.31 | 5.16E-05 | 3.07E-03 |
| autophagy of mitochondrion (GO:0000422) | 43 | 4 | 0.19 | + | 21.31 | 5.16E-05 | 3.08E-03 |
| regulation of peptidyl-serine phosphorylation (GO:0033135) | 146 | 6 | 0.64 | + | 9.42 | 5.31E-05 | 3.13E-03 |
| neuromuscular junction development (GO:0007528) | 44 | 4 | 0.19 | + | 20.83 | 5.61E-05 | 3.29E-03 |
| vacuolar transport (GO:0007034) | 149 | 6 | 0.65 | + | 9.23 | 5.92E-05 | 3.46E-03 |
| membrane organization (GO:0061024) | 939 | 14 | 4.1 | + | 3.42 | 6.04E-05 | 3.52E-03 |
| prepulse inhibition (GO:0060134) | 15 | 3 | 0.07 | + | 45.83 | 6.18E-05 | 3.58E-03 |
| regulation of protein targeting to mitochondrion (GO:1903214) | 46 | 4 | 0.2 | + | 19.92 | 6.59E-05 | 3.80E-03 |
| positive regulation of small molecule metabolic process (GO:0062013) | 152 | 6 | 0.66 | + | 9.04 | 6.59E-05 | 3.81E-03 |
| negative regulation of neuron projection development (GO:0010977) | 153 | 6 | 0.67 | + | 8.99 | 6.82E-05 | 3.90E-03 |
| sphingolipid biosynthetic process (GO:0030148) | 93 | 5 | 0.41 | + | 12.32 | 6.85E-05 | 3.90E-03 |
| ion transport (GO:0006811) | 1343 | 17 | 5.86 | + | 2.9 | 6.87E-05 | 3.90E-03 |
| negative regulation of establishment of protein localization (GO:1904950) | 153 | 6 | 0.67 | + | 8.99 | 6.82E-05 | 3.92E-03 |
| regulation of protein deacetylation (GO:0090311) | 47 | 4 | 0.21 | + | 19.5 | 7.13E-05 | 4.03E-03 |
| organonitrogen compound catabolic process (GO:1901565) | 1083 | 15 | 4.73 | + | 3.17 | 7.30E-05 | 4.12E-03 |
| cation transport (GO:0006812) | 839 | 13 | 3.66 | + | 3.55 | 7.78E-05 | 4.37E-03 |
| response to nitrogen compound (GO:1901698) | 1093 | 15 | 4.77 | + | 3.14 | 8.09E-05 | 4.53E-03 |
| positive regulation of protein catabolic process (GO:0045732) | 232 | 7 | 1.01 | + | 6.91 | 8.28E-05 | 4.62E-03 |
| positive regulation of phosphorylation (GO:0042327) | 1096 | 15 | 4.78 | + | 3.14 | 8.33E-05 | 4.63E-03 |
| regulation of cell morphogenesis involved in differentiation (GO:0010769) | 316 | 8 | 1.38 | + | 5.8 | 8.40E-05 | 4.65E-03 |
| positive regulation of mitochondrial fission (GO:0090141) | 17 | 3 | 0.07 | + | 40.44 | 8.58E-05 | 4.73E-03 |
| negative regulation of intrinsic apoptotic signaling pathway (GO:2001243) | 98 | 5 | 0.43 | + | 11.69 | 8.68E-05 | 4.77E-03 |
| regulation of MAPK cascade (GO:0043408) | 732 | 12 | 3.19 | + | 3.76 | 8.92E-05 | 4.89E-03 |
| regulation of calcium-mediated signaling (GO:0050848) | 99 | 5 | 0.43 | + | 11.57 | 9.09E-05 | 4.96E-03 |
| regulation of cell development (GO:0060284) | 977 | 14 | 4.26 | + | 3.28 | 9.17E-05 | 4.99E-03 |
| regulation of ubiquitin-dependent protein catabolic process (GO:2000058) | 162 | 6 | 0.71 | + | 8.49 | 9.25E-05 | 5.02E-03 |
| organic substance transport (GO:0071702) | 2218 | 23 | 9.68 | + | 2.38 | 9.81E-05 | 5.30E-03 |
| regulation of peptide secretion (GO:0002791) | 417 | 9 | 1.82 | + | 4.95 | 9.85E-05 | 5.31E-03 |
| synaptic vesicle endocytosis (GO:0048488) | 52 | 4 | 0.23 | + | 17.63 | 1.03E-04 | 5.52E-03 |
| presynaptic endocytosis (GO:0140238) | 52 | 4 | 0.23 | + | 17.63 | 1.03E-04 | 5.54E-03 |
| positive regulation of reactive oxygen species metabolic process (GO:2000379) | 102 | 5 | 0.45 | + | 11.23 | 1.04E-04 | 5.55E-03 |
| negative regulation of late endosome to lysosome transport (GO:1902823) | 2 | 2 | 0.01 | + | > 100 | 1.11E-04 | 5.79E-03 |
| negative regulation of vacuolar transport (GO:1903336) | 2 | 2 | 0.01 | + | > 100 | 1.11E-04 | 5.81E-03 |
| regulation of multicellular organismal process (GO:0051239) | 3242 | 29 | 14.15 | + | 2.05 | 1.12E-04 | 5.81E-03 |
| regulation of hydrogen peroxide-induced neuron intrinsic apoptotic signaling pathway (GO:1903383) | 2 | 2 | 0.01 | + | > 100 | 1.11E-04 | 5.83E-03 |
| negative regulation of hydrogen peroxide-induced neuron intrinsic apoptotic signaling pathway (GO:1903384) | 2 | 2 | 0.01 | + | > 100 | 1.11E-04 | 5.85E-03 |
| positive regulation of ubiquitin-dependent protein catabolic process (GO:2000060) | 104 | 5 | 0.45 | + | 11.02 | 1.13E-04 | 5.86E-03 |
| regulation of presynaptic membrane organization (GO:1901629) | 2 | 2 | 0.01 | + | > 100 | 1.11E-04 | 5.87E-03 |
| positive regulation of presynaptic membrane organization (GO:1901631) | 2 | 2 | 0.01 | + | > 100 | 1.11E-04 | 5.89E-03 |
| positive regulation of histone deacetylation (GO:0031065) | 19 | 3 | 0.08 | + | 36.18 | 1.15E-04 | 5.89E-03 |
| astrocyte activation (GO:0048143) | 19 | 3 | 0.08 | + | 36.18 | 1.15E-04 | 5.90E-03 |
| regulation of dopamine biosynthetic process (GO:1903179) | 2 | 2 | 0.01 | + | > 100 | 1.11E-04 | 5.90E-03 |
| neurogenesis (GO:0022008) | 1690 | 19 | 7.38 | + | 2.58 | 1.16E-04 | 5.91E-03 |
| postsynapse assembly (GO:0099068) | 19 | 3 | 0.08 | + | 36.18 | 1.15E-04 | 5.92E-03 |
| positive regulation of dopamine biosynthetic process (GO:1903181) | 2 | 2 | 0.01 | + | > 100 | 1.11E-04 | 5.92E-03 |
| positive regulation of endocytosis (GO:0045807) | 105 | 5 | 0.46 | + | 10.91 | 1.18E-04 | 6.02E-03 |
| positive regulation of protein-containing complex assembly (GO:0031334) | 249 | 7 | 1.09 | + | 6.44 | 1.27E-04 | 6.37E-03 |
| positive regulation of oxidoreductase activity (GO:0051353) | 55 | 4 | 0.24 | + | 16.66 | 1.26E-04 | 6.38E-03 |
| negative regulation of cell communication (GO:0010648) | 1413 | 17 | 6.17 | + | 2.76 | 1.27E-04 | 6.39E-03 |
| regulation of ubiquitin-protein transferase activity (GO:0051438) | 55 | 4 | 0.24 | + | 16.66 | 1.26E-04 | 6.40E-03 |
| negative regulation of signaling (GO:0023057) | 1416 | 17 | 6.18 | + | 2.75 | 1.30E-04 | 6.51E-03 |
| negative regulation of endoplasmic reticulum stress-induced intrinsic apoptotic signaling pathway (GO:1902236) | 20 | 3 | 0.09 | + | 34.37 | 1.32E-04 | 6.58E-03 |
| lysosomal transport (GO:0007041) | 108 | 5 | 0.47 | + | 10.61 | 1.35E-04 | 6.68E-03 |
| alcohol catabolic process (GO:0046164) | 56 | 4 | 0.24 | + | 16.37 | 1.35E-04 | 6.69E-03 |
| positive regulation of cellular component biogenesis (GO:0044089) | 541 | 10 | 2.36 | + | 4.24 | 1.39E-04 | 6.86E-03 |
| organic hydroxy compound biosynthetic process (GO:1901617) | 175 | 6 | 0.76 | + | 7.86 | 1.39E-04 | 6.87E-03 |
| positive regulation of protein phosphorylation (GO:0001934) | 1019 | 14 | 4.45 | + | 3.15 | 1.42E-04 | 6.96E-03 |
| positive regulation of apoptotic process (GO:0043065) | 655 | 11 | 2.86 | + | 3.85 | 1.46E-04 | 7.13E-03 |
| positive regulation of binding (GO:0051099) | 177 | 6 | 0.77 | + | 7.77 | 1.48E-04 | 7.19E-03 |
| regulation of response to stimulus (GO:0048583) | 4382 | 35 | 19.12 | + | 1.83 | 1.48E-04 | 7.20E-03 |
| positive regulation of cellular protein localization (GO:1903829) | 344 | 8 | 1.5 | + | 5.33 | 1.49E-04 | 7.21E-03 |
| central nervous system development (GO:0007417) | 1024 | 14 | 4.47 | + | 3.13 | 1.49E-04 | 7.21E-03 |
| response to endoplasmic reticulum stress (GO:0034976) | 256 | 7 | 1.12 | + | 6.27 | 1.50E-04 | 7.23E-03 |
| positive regulation of peptidyl-serine phosphorylation (GO:0033138) | 111 | 5 | 0.48 | + | 10.32 | 1.52E-04 | 7.31E-03 |
| regulation of monooxygenase activity (GO:0032768) | 58 | 4 | 0.25 | + | 15.8 | 1.53E-04 | 7.35E-03 |
| protein metabolic process (GO:0019538) | 4414 | 35 | 19.26 | + | 1.82 | 1.62E-04 | 7.74E-03 |
| cellular response to toxic substance (GO:0097237) | 113 | 5 | 0.49 | + | 10.14 | 1.65E-04 | 7.85E-03 |
| positive regulation of secretion by cell (GO:1903532) | 350 | 8 | 1.53 | + | 5.24 | 1.67E-04 | 7.92E-03 |
| synaptic vesicle recycling (GO:0036465) | 60 | 4 | 0.26 | + | 15.28 | 1.74E-04 | 8.19E-03 |
| positive regulation of nervous system development (GO:0051962) | 556 | 10 | 2.43 | + | 4.12 | 1.73E-04 | 8.20E-03 |
| negative regulation of cell projection organization (GO:0031345) | 183 | 6 | 0.8 | + | 7.51 | 1.76E-04 | 8.28E-03 |
| positive regulation of mitochondrial electron transport, NADH to ubiquinone (GO:1902958) | 3 | 2 | 0.01 | + | > 100 | 1.85E-04 | 8.53E-03 |
| termination of signal transduction (GO:0023021) | 3 | 2 | 0.01 | + | > 100 | 1.85E-04 | 8.56E-03 |
| regulation of intrinsic apoptotic signaling pathway in response to hydrogen peroxide (GO:1903750) | 3 | 2 | 0.01 | + | > 100 | 1.85E-04 | 8.58E-03 |
| negative regulation of intrinsic apoptotic signaling pathway in response to hydrogen peroxide (GO:1903751) | 3 | 2 | 0.01 | + | > 100 | 1.85E-04 | 8.61E-03 |
| regulation of peroxidase activity (GO:2000468) | 3 | 2 | 0.01 | + | > 100 | 1.85E-04 | 8.63E-03 |
| cellular metabolic process (GO:0044237) | 7784 | 52 | 33.97 | + | 1.53 | 1.85E-04 | 8.64E-03 |
| modulation by host of symbiont process (GO:0051851) | 61 | 4 | 0.27 | + | 15.03 | 1.84E-04 | 8.65E-03 |
| regulation of peptidase activity (GO:0052547) | 456 | 9 | 1.99 | + | 4.52 | 1.90E-04 | 8.74E-03 |
| presynapse assembly (GO:0099054) | 23 | 3 | 0.1 | + | 29.89 | 1.92E-04 | 8.77E-03 |
| cellular response to stimulus (GO:0051716) | 6808 | 47 | 29.71 | + | 1.58 | 1.93E-04 | 8.79E-03 |
| positive regulation of protein deacetylation (GO:0090312) | 23 | 3 | 0.1 | + | 29.89 | 1.92E-04 | 8.79E-03 |
| negative regulation of cellular protein localization (GO:1903828) | 119 | 5 | 0.52 | + | 9.63 | 2.08E-04 | 9.44E-03 |
| regulation of axonogenesis (GO:0050770) | 192 | 6 | 0.84 | + | 7.16 | 2.27E-04 | 1.03E-02 |
| aging (GO:0007568) | 279 | 7 | 1.22 | + | 5.75 | 2.50E-04 | 1.13E-02 |
| regulation of response to external stimulus (GO:0032101) | 1213 | 15 | 5.29 | + | 2.83 | 2.50E-04 | 1.13E-02 |
| polyol metabolic process (GO:0019751) | 124 | 5 | 0.54 | + | 9.24 | 2.50E-04 | 1.13E-02 |
| cellular response to metal ion (GO:0071248) | 196 | 6 | 0.86 | + | 7.01 | 2.52E-04 | 1.13E-02 |
| autophagosome organization (GO:1905037) | 67 | 4 | 0.29 | + | 13.68 | 2.60E-04 | 1.16E-02 |
| regulation of cytoplasmic transport (GO:1903649) | 26 | 3 | 0.11 | + | 26.44 | 2.67E-04 | 1.19E-02 |
| presynapse organization (GO:0099172) | 26 | 3 | 0.11 | + | 26.44 | 2.67E-04 | 1.19E-02 |
| system process (GO:0003008) | 2072 | 21 | 9.04 | + | 2.32 | 2.69E-04 | 1.19E-02 |
| regulation of late endosome to lysosome transport (GO:1902822) | 4 | 2 | 0.02 | + | > 100 | 2.77E-04 | 1.20E-02 |
| positive regulation of secretion (GO:0051047) | 378 | 8 | 1.65 | + | 4.85 | 2.78E-04 | 1.20E-02 |
| negative regulation of protein catabolic process in the vacuole (GO:1904351) | 4 | 2 | 0.02 | + | > 100 | 2.77E-04 | 1.20E-02 |
| aminergic neurotransmitter loading into synaptic vesicle (GO:0015842) | 4 | 2 | 0.02 | + | > 100 | 2.77E-04 | 1.20E-02 |
| negative regulation of lysosomal protein catabolic process (GO:1905166) | 4 | 2 | 0.02 | + | > 100 | 2.77E-04 | 1.21E-02 |
| regulation of endosome organization (GO:1904978) | 4 | 2 | 0.02 | + | > 100 | 2.77E-04 | 1.21E-02 |
| negative regulation of mitochondrial fission (GO:0090258) | 4 | 2 | 0.02 | + | > 100 | 2.77E-04 | 1.21E-02 |
| positive regulation of amyloid fibril formation (GO:1905908) | 4 | 2 | 0.02 | + | > 100 | 2.77E-04 | 1.22E-02 |
| astrocyte activation involved in immune response (GO:0002265) | 4 | 2 | 0.02 | + | > 100 | 2.77E-04 | 1.22E-02 |
| regulation of TRAIL-activated apoptotic signaling pathway (GO:1903121) | 4 | 2 | 0.02 | + | > 100 | 2.77E-04 | 1.22E-02 |
| macromolecule localization (GO:0033036) | 2551 | 24 | 11.13 | + | 2.16 | 2.85E-04 | 1.22E-02 |
| lytic vacuole organization (GO:0080171) | 69 | 4 | 0.3 | + | 13.28 | 2.89E-04 | 1.23E-02 |
| positive regulation of proteolysis involved in cellular protein catabolic process (GO:1903052) | 128 | 5 | 0.56 | + | 8.95 | 2.88E-04 | 1.23E-02 |
| lysosome organization (GO:0007040) | 69 | 4 | 0.3 | + | 13.28 | 2.89E-04 | 1.23E-02 |
| positive regulation of ion transport (GO:0043270) | 287 | 7 | 1.25 | + | 5.59 | 2.96E-04 | 1.25E-02 |
| regulation of axon regeneration (GO:0048679) | 27 | 3 | 0.12 | + | 25.46 | 2.96E-04 | 1.25E-02 |
| steroid catabolic process (GO:0006706) | 27 | 3 | 0.12 | + | 25.46 | 2.96E-04 | 1.26E-02 |
| positive regulation of neuron differentiation (GO:0045666) | 383 | 8 | 1.67 | + | 4.79 | 3.03E-04 | 1.28E-02 |
| positive regulation of neurogenesis (GO:0050769) | 489 | 9 | 2.13 | + | 4.22 | 3.15E-04 | 1.33E-02 |
| signaling (GO:0023052) | 5518 | 40 | 24.08 | + | 1.66 | 3.17E-04 | 1.33E-02 |
| sterol transport (GO:0015918) | 71 | 4 | 0.31 | + | 12.91 | 3.20E-04 | 1.33E-02 |
| multicellular organismal response to stress (GO:0033555) | 71 | 4 | 0.31 | + | 12.91 | 3.20E-04 | 1.33E-02 |
| regulation of protein secretion (GO:0050708) | 386 | 8 | 1.68 | + | 4.75 | 3.19E-04 | 1.33E-02 |
| response to lipid (GO:0033993) | 842 | 12 | 3.67 | + | 3.27 | 3.20E-04 | 1.33E-02 |
| lipid biosynthetic process (GO:0008610) | 602 | 10 | 2.63 | + | 3.81 | 3.24E-04 | 1.34E-02 |
| reproductive behavior (GO:0019098) | 28 | 3 | 0.12 | + | 24.55 | 3.27E-04 | 1.35E-02 |
| regulation of proteasomal ubiquitin-dependent protein catabolic process (GO:0032434) | 132 | 5 | 0.58 | + | 8.68 | 3.30E-04 | 1.36E-02 |
| organic substance metabolic process (GO:0071704) | 8007 | 52 | 34.94 | + | 1.49 | 3.30E-04 | 1.36E-02 |
| cellular response to organonitrogen compound (GO:0071417) | 604 | 10 | 2.64 | + | 3.79 | 3.32E-04 | 1.36E-02 |
| negative regulation of axonogenesis (GO:0050771) | 72 | 4 | 0.31 | + | 12.73 | 3.37E-04 | 1.38E-02 |
| regulation of anatomical structure morphogenesis (GO:0022603) | 1110 | 14 | 4.84 | + | 2.89 | 3.38E-04 | 1.38E-02 |
| negative regulation of protein catabolic process (GO:0042177) | 133 | 5 | 0.58 | + | 8.61 | 3.41E-04 | 1.38E-02 |
| regulation of protein localization to nucleus (GO:1900180) | 133 | 5 | 0.58 | + | 8.61 | 3.41E-04 | 1.39E-02 |
| cellular protein metabolic process (GO:0044267) | 3826 | 31 | 16.7 | + | 1.86 | 3.44E-04 | 1.39E-02 |
| positive regulation of protein transport (GO:0051222) | 392 | 8 | 1.71 | + | 4.68 | 3.53E-04 | 1.42E-02 |
| regulation of endocytosis (GO:0030100) | 209 | 6 | 0.91 | + | 6.58 | 3.52E-04 | 1.42E-02 |
| modulation by host of viral process (GO:0044788) | 29 | 3 | 0.13 | + | 23.7 | 3.59E-04 | 1.44E-02 |
| regulation of protein ubiquitination (GO:0031396) | 210 | 6 | 0.92 | + | 6.55 | 3.61E-04 | 1.45E-02 |
| transition metal ion homeostasis (GO:0055076) | 135 | 5 | 0.59 | + | 8.49 | 3.64E-04 | 1.46E-02 |
| regulation of vacuolar transport (GO:1903335) | 5 | 2 | 0.02 | + | 91.65 | 3.87E-04 | 1.51E-02 |
| organonitrogen compound biosynthetic process (GO:1901566) | 1405 | 16 | 6.13 | + | 2.61 | 3.80E-04 | 1.51E-02 |
| synaptic vesicle uncoating (GO:0016191) | 5 | 2 | 0.02 | + | 91.65 | 3.87E-04 | 1.51E-02 |
| positive regulation of neuromuscular junction development (GO:1904398) | 5 | 2 | 0.02 | + | 91.65 | 3.87E-04 | 1.52E-02 |
| negative regulation of MAP kinase activity (GO:0043407) | 75 | 4 | 0.33 | + | 12.22 | 3.91E-04 | 1.52E-02 |
| regulation of cellular respiration (GO:0043457) | 30 | 3 | 0.13 | + | 22.91 | 3.94E-04 | 1.52E-02 |
| regulation of retrograde transport, endosome to Golgi (GO:1905279) | 5 | 2 | 0.02 | + | 91.65 | 3.87E-04 | 1.52E-02 |
| organelle disassembly (GO:1903008) | 75 | 4 | 0.33 | + | 12.22 | 3.91E-04 | 1.52E-02 |
| regulation of neuron projection regeneration (GO:0070570) | 30 | 3 | 0.13 | + | 22.91 | 3.94E-04 | 1.52E-02 |
| positive regulation of mitochondrial ATP synthesis coupled electron transport (GO:1905448) | 5 | 2 | 0.02 | + | 91.65 | 3.87E-04 | 1.53E-02 |
| postsynaptic membrane organization (GO:0001941) | 30 | 3 | 0.13 | + | 22.91 | 3.94E-04 | 1.53E-02 |
| negative regulation of hydrogen peroxide-mediated programmed cell death (GO:1901299) | 5 | 2 | 0.02 | + | 91.65 | 3.87E-04 | 1.53E-02 |
| regulation of ubiquitin-specific protease activity (GO:2000152) | 5 | 2 | 0.02 | + | 91.65 | 3.87E-04 | 1.53E-02 |
| positive regulation of dopamine receptor signaling pathway (GO:0060161) | 5 | 2 | 0.02 | + | 91.65 | 3.87E-04 | 1.54E-02 |
| response to nutrient levels (GO:0031667) | 506 | 9 | 2.21 | + | 4.08 | 4.02E-04 | 1.55E-02 |
| negative regulation of nitrogen compound metabolic process (GO:0051172) | 2425 | 23 | 10.58 | + | 2.17 | 4.04E-04 | 1.55E-02 |
| regulation of apoptotic process (GO:0042981) | 1561 | 17 | 6.81 | + | 2.5 | 4.07E-04 | 1.56E-02 |
| regulation of cell morphogenesis (GO:0022604) | 508 | 9 | 2.22 | + | 4.06 | 4.14E-04 | 1.58E-02 |
| regulation of signaling (GO:0023051) | 3630 | 30 | 15.84 | + | 1.89 | 4.18E-04 | 1.59E-02 |
| negative regulation of reactive oxygen species biosynthetic process (GO:1903427) | 31 | 3 | 0.14 | + | 22.17 | 4.31E-04 | 1.62E-02 |
| neurotransmitter metabolic process (GO:0042133) | 31 | 3 | 0.14 | + | 22.17 | 4.31E-04 | 1.63E-02 |
| regulation of histone deacetylation (GO:0031063) | 31 | 3 | 0.14 | + | 22.17 | 4.31E-04 | 1.63E-02 |
| regulation of oxidative phosphorylation (GO:0002082) | 31 | 3 | 0.14 | + | 22.17 | 4.31E-04 | 1.63E-02 |
| positive regulation of monooxygenase activity (GO:0032770) | 31 | 3 | 0.14 | + | 22.17 | 4.31E-04 | 1.64E-02 |
| lipid transport (GO:0006869) | 308 | 7 | 1.34 | + | 5.21 | 4.48E-04 | 1.68E-02 |
| positive regulation of establishment of protein localization (GO:1904951) | 408 | 8 | 1.78 | + | 4.49 | 4.57E-04 | 1.72E-02 |
| regulation of apoptotic signaling pathway (GO:2001233) | 409 | 8 | 1.78 | + | 4.48 | 4.65E-04 | 1.74E-02 |
| microglial cell activation (GO:0001774) | 32 | 3 | 0.14 | + | 21.48 | 4.70E-04 | 1.75E-02 |
| leukocyte activation involved in inflammatory response (GO:0002269) | 32 | 3 | 0.14 | + | 21.48 | 4.70E-04 | 1.75E-02 |
| regulation of programmed cell death (GO:0043067) | 1583 | 17 | 6.91 | + | 2.46 | 4.77E-04 | 1.77E-02 |
| protein localization to mitochondrion (GO:0070585) | 80 | 4 | 0.35 | + | 11.46 | 4.94E-04 | 1.83E-02 |
| negative regulation of signal transduction (GO:0009968) | 1295 | 15 | 5.65 | + | 2.65 | 4.97E-04 | 1.84E-02 |
| cellular response to inorganic substance (GO:0071241) | 224 | 6 | 0.98 | + | 6.14 | 5.04E-04 | 1.86E-02 |
| mitochondrial transport (GO:0006839) | 225 | 6 | 0.98 | + | 6.11 | 5.15E-04 | 1.87E-02 |
| positive regulation of catecholamine metabolic process (GO:0045915) | 6 | 2 | 0.03 | + | 76.38 | 5.14E-04 | 1.87E-02 |
| diol metabolic process (GO:0034311) | 33 | 3 | 0.14 | + | 20.83 | 5.11E-04 | 1.87E-02 |
| positive regulation of dopamine metabolic process (GO:0045964) | 6 | 2 | 0.03 | + | 76.38 | 5.14E-04 | 1.87E-02 |
| negative regulation of oxidoreductase activity (GO:0051354) | 33 | 3 | 0.14 | + | 20.83 | 5.11E-04 | 1.88E-02 |
| clathrin coat disassembly (GO:0072318) | 6 | 2 | 0.03 | + | 76.38 | 5.14E-04 | 1.88E-02 |
| regulation of endoplasmic reticulum stress-induced intrinsic apoptotic signaling pathway (GO:1902235) | 33 | 3 | 0.14 | + | 20.83 | 5.11E-04 | 1.88E-02 |
| negative regulation of neuron differentiation (GO:0045665) | 226 | 6 | 0.99 | + | 6.08 | 5.27E-04 | 1.91E-02 |
| positive regulation of molecular function (GO:0044093) | 1837 | 19 | 8.02 | + | 2.37 | 5.47E-04 | 1.98E-02 |
| learning (GO:0007612) | 149 | 5 | 0.65 | + | 7.69 | 5.63E-04 | 2.03E-02 |
| negative regulation of protein transport (GO:0051224) | 149 | 5 | 0.65 | + | 7.69 | 5.63E-04 | 2.03E-02 |
| nitrogen compound transport (GO:0071705) | 1857 | 19 | 8.1 | + | 2.34 | 5.76E-04 | 2.07E-02 |
| dephosphorylation (GO:0016311) | 322 | 7 | 1.41 | + | 4.98 | 5.80E-04 | 2.08E-02 |
| regulation of protein targeting (GO:1903533) | 84 | 4 | 0.37 | + | 10.91 | 5.89E-04 | 2.10E-02 |
| negative regulation of macroautophagy (GO:0016242) | 35 | 3 | 0.15 | + | 19.64 | 6.00E-04 | 2.13E-02 |
| regulation of presynapse assembly (GO:1905606) | 35 | 3 | 0.15 | + | 19.64 | 6.00E-04 | 2.13E-02 |
| negative regulation of apoptotic signaling pathway (GO:2001234) | 232 | 6 | 1.01 | + | 5.93 | 6.03E-04 | 2.13E-02 |
| regulation of dopamine secretion (GO:0014059) | 35 | 3 | 0.15 | + | 19.64 | 6.00E-04 | 2.14E-02 |
| positive regulation of metabolic process (GO:0009893) | 3907 | 31 | 17.05 | + | 1.82 | 6.24E-04 | 2.20E-02 |
| response to extracellular stimulus (GO:0009991) | 540 | 9 | 2.36 | + | 3.82 | 6.37E-04 | 2.25E-02 |
| negative regulation of cellular protein catabolic process (GO:1903363) | 86 | 4 | 0.38 | + | 10.66 | 6.41E-04 | 2.25E-02 |
| amyloid-beta clearance by cellular catabolic process (GO:0150094) | 7 | 2 | 0.03 | + | 65.47 | 6.59E-04 | 2.27E-02 |
| regulation of presynapse organization (GO:0099174) | 36 | 3 | 0.16 | + | 19.09 | 6.48E-04 | 2.27E-02 |
| response to herbicide (GO:0009635) | 7 | 2 | 0.03 | + | 65.47 | 6.59E-04 | 2.27E-02 |
| membrane assembly (GO:0071709) | 36 | 3 | 0.16 | + | 19.09 | 6.48E-04 | 2.27E-02 |
| regulation of lipid localization (GO:1905952) | 154 | 5 | 0.67 | + | 7.44 | 6.51E-04 | 2.28E-02 |
| voluntary musculoskeletal movement (GO:0050882) | 7 | 2 | 0.03 | + | 65.47 | 6.59E-04 | 2.28E-02 |
| cellular response to organic substance (GO:0071310) | 2360 | 22 | 10.3 | + | 2.14 | 6.55E-04 | 2.28E-02 |
| negative regulation by host of viral genome replication (GO:0044828) | 7 | 2 | 0.03 | + | 65.47 | 6.59E-04 | 2.28E-02 |
| positive regulation of catalytic activity (GO:0043085) | 1478 | 16 | 6.45 | + | 2.48 | 6.57E-04 | 2.29E-02 |
| vesicle uncoating (GO:0072319) | 7 | 2 | 0.03 | + | 65.47 | 6.59E-04 | 2.29E-02 |
| cellular response to nitrogen compound (GO:1901699) | 662 | 10 | 2.89 | + | 3.46 | 6.71E-04 | 2.30E-02 |
| regulation of G1/S transition of mitotic cell cycle (GO:2000045) | 155 | 5 | 0.68 | + | 7.39 | 6.70E-04 | 2.30E-02 |
| regulation of cell communication (GO:0010646) | 3591 | 29 | 15.67 | + | 1.85 | 6.90E-04 | 2.35E-02 |
| regulation of histone modification (GO:0031056) | 156 | 5 | 0.68 | + | 7.34 | 6.89E-04 | 2.36E-02 |
| positive regulation of protein binding (GO:0032092) | 88 | 4 | 0.38 | + | 10.42 | 6.96E-04 | 2.37E-02 |
| zinc ion homeostasis (GO:0055069) | 37 | 3 | 0.16 | + | 18.58 | 6.98E-04 | 2.37E-02 |
| positive regulation of proteasomal ubiquitin-dependent protein catabolic process (GO:0032436) | 88 | 4 | 0.38 | + | 10.42 | 6.96E-04 | 2.37E-02 |
| positive regulation of nitrogen compound metabolic process (GO:0051173) | 3249 | 27 | 14.18 | + | 1.9 | 7.05E-04 | 2.38E-02 |
| regulation of dendrite development (GO:0050773) | 157 | 5 | 0.69 | + | 7.3 | 7.09E-04 | 2.39E-02 |
| interaction with symbiont (GO:0051702) | 89 | 4 | 0.39 | + | 10.3 | 7.25E-04 | 2.44E-02 |
| regulation of protein modification by small protein conjugation or removal (GO:1903320) | 241 | 6 | 1.05 | + | 5.7 | 7.32E-04 | 2.46E-02 |
| cellular response to amyloid-beta (GO:1904646) | 38 | 3 | 0.17 | + | 18.09 | 7.51E-04 | 2.51E-02 |
| astrocyte development (GO:0014002) | 38 | 3 | 0.17 | + | 18.09 | 7.51E-04 | 2.52E-02 |
| post-embryonic development (GO:0009791) | 90 | 4 | 0.39 | + | 10.18 | 7.55E-04 | 2.52E-02 |
| regulation of cellular metabolic process (GO:0031323) | 6267 | 43 | 27.35 | + | 1.57 | 7.82E-04 | 2.61E-02 |
| postsynapse organization (GO:0099173) | 91 | 4 | 0.4 | + | 10.07 | 7.85E-04 | 2.61E-02 |
| lipid localization (GO:0010876) | 340 | 7 | 1.48 | + | 4.72 | 7.94E-04 | 2.63E-02 |
| negative regulation of biological process (GO:0048519) | 5557 | 39 | 24.25 | + | 1.61 | 8.22E-04 | 2.68E-02 |
| dopamine catabolic process (GO:0042420) | 8 | 2 | 0.03 | + | 57.28 | 8.22E-04 | 2.68E-02 |
| amine transport (GO:0015837) | 8 | 2 | 0.03 | + | 57.28 | 8.22E-04 | 2.69E-02 |
| positive regulation of ion transmembrane transport (GO:0034767) | 162 | 5 | 0.71 | + | 7.07 | 8.13E-04 | 2.69E-02 |
| regulation of chaperone-mediated autophagy (GO:1904714) | 8 | 2 | 0.03 | + | 57.28 | 8.22E-04 | 2.69E-02 |
| intracellular distribution of mitochondria (GO:0048312) | 8 | 2 | 0.03 | + | 57.28 | 8.22E-04 | 2.70E-02 |
| neurotransmitter loading into synaptic vesicle (GO:0098700) | 8 | 2 | 0.03 | + | 57.28 | 8.22E-04 | 2.70E-02 |
| regulation of intrinsic apoptotic signaling pathway (GO:2001242) | 163 | 5 | 0.71 | + | 7.03 | 8.35E-04 | 2.71E-02 |
| hyaloid vascular plexus regression (GO:1990384) | 8 | 2 | 0.03 | + | 57.28 | 8.22E-04 | 2.71E-02 |
| regulation of developmental growth (GO:0048638) | 343 | 7 | 1.5 | + | 4.68 | 8.35E-04 | 2.71E-02 |
| inclusion body assembly (GO:0070841) | 8 | 2 | 0.03 | + | 57.28 | 8.22E-04 | 2.72E-02 |
| lipoprotein biosynthetic process (GO:0042158) | 93 | 4 | 0.41 | + | 9.86 | 8.49E-04 | 2.74E-02 |
| regulation of amine transport (GO:0051952) | 93 | 4 | 0.41 | + | 9.86 | 8.49E-04 | 2.75E-02 |
| positive regulation of cell development (GO:0010720) | 567 | 9 | 2.47 | + | 3.64 | 8.95E-04 | 2.89E-02 |
| positive regulation of cellular metabolic process (GO:0031325) | 3432 | 28 | 14.98 | + | 1.87 | 9.17E-04 | 2.95E-02 |
| positive regulation of lipid localization (GO:1905954) | 95 | 4 | 0.41 | + | 9.65 | 9.17E-04 | 2.95E-02 |
| primary metabolic process (GO:0044238) | 7572 | 49 | 33.05 | + | 1.48 | 9.38E-04 | 3.01E-02 |
| maintenance of location in cell (GO:0051651) | 96 | 4 | 0.42 | + | 9.55 | 9.52E-04 | 3.04E-02 |
| regulation of small molecule metabolic process (GO:0062012) | 458 | 8 | 2 | + | 4 | 9.58E-04 | 3.06E-02 |
| positive regulation of multicellular organismal process (GO:0051240) | 1794 | 18 | 7.83 | + | 2.3 | 9.67E-04 | 3.08E-02 |
| membrane biogenesis (GO:0044091) | 42 | 3 | 0.18 | + | 16.37 | 9.87E-04 | 3.14E-02 |
| negative regulation of establishment of protein localization to mitochondrion (GO:1903748) | 9 | 2 | 0.04 | + | 50.92 | 1.00E-03 | 3.14E-02 |
| positive regulation of mitophagy in response to mitochondrial depolarization (GO:0098779) | 9 | 2 | 0.04 | + | 50.92 | 1.00E-03 | 3.15E-02 |
| regulation of hydrogen peroxide-mediated programmed cell death (GO:1901298) | 9 | 2 | 0.04 | + | 50.92 | 1.00E-03 | 3.15E-02 |
| regulation of amyloid fibril formation (GO:1905906) | 9 | 2 | 0.04 | + | 50.92 | 1.00E-03 | 3.16E-02 |
| mitochondrial DNA replication (GO:0006264) | 9 | 2 | 0.04 | + | 50.92 | 1.00E-03 | 3.16E-02 |
| steroid metabolic process (GO:0008202) | 257 | 6 | 1.12 | + | 5.35 | 1.01E-03 | 3.17E-02 |
| regulation of resting membrane potential (GO:0060075) | 9 | 2 | 0.04 | + | 50.92 | 1.00E-03 | 3.17E-02 |
| negative regulation of autophagy of mitochondrion (GO:1903147) | 9 | 2 | 0.04 | + | 50.92 | 1.00E-03 | 3.18E-02 |
| negative regulation of intracellular protein transport (GO:0090317) | 43 | 3 | 0.19 | + | 15.99 | 1.05E-03 | 3.28E-02 |
| secretion (GO:0046903) | 1104 | 13 | 4.82 | + | 2.7 | 1.05E-03 | 3.29E-02 |
| positive regulation of neuron death (GO:1901216) | 99 | 4 | 0.43 | + | 9.26 | 1.06E-03 | 3.31E-02 |
| negative regulation of cell morphogenesis involved in differentiation (GO:0010771) | 100 | 4 | 0.44 | + | 9.17 | 1.10E-03 | 3.41E-02 |
| regulation of cyclin-dependent protein serine/threonine kinase activity (GO:0000079) | 100 | 4 | 0.44 | + | 9.17 | 1.10E-03 | 3.41E-02 |
| maintenance of protein location (GO:0045185) | 100 | 4 | 0.44 | + | 9.17 | 1.10E-03 | 3.42E-02 |
| positive regulation of dendritic spine development (GO:0060999) | 44 | 3 | 0.19 | + | 15.62 | 1.12E-03 | 3.44E-02 |
| positive regulation of protein dephosphorylation (GO:0035307) | 44 | 3 | 0.19 | + | 15.62 | 1.12E-03 | 3.45E-02 |
| multicellular organismal movement (GO:0050879) | 44 | 3 | 0.19 | + | 15.62 | 1.12E-03 | 3.45E-02 |
| musculoskeletal movement (GO:0050881) | 44 | 3 | 0.19 | + | 15.62 | 1.12E-03 | 3.46E-02 |
| regulation of cell cycle G1/S phase transition (GO:1902806) | 175 | 5 | 0.76 | + | 6.55 | 1.14E-03 | 3.48E-02 |
| regulation of ion transport (GO:0043269) | 711 | 10 | 3.1 | + | 3.22 | 1.15E-03 | 3.50E-02 |
| protein modification process (GO:0036211) | 3192 | 26 | 13.93 | + | 1.87 | 1.17E-03 | 3.55E-02 |
| regulation of response to wounding (GO:1903034) | 176 | 5 | 0.77 | + | 6.51 | 1.16E-03 | 3.55E-02 |
| cellular protein modification process (GO:0006464) | 3192 | 26 | 13.93 | + | 1.87 | 1.17E-03 | 3.56E-02 |
| positive regulation of developmental process (GO:0051094) | 1410 | 15 | 6.15 | + | 2.44 | 1.18E-03 | 3.57E-02 |
| catechol-containing compound catabolic process (GO:0019614) | 10 | 2 | 0.04 | + | 45.83 | 1.20E-03 | 3.59E-02 |
| intestinal cholesterol absorption (GO:0030299) | 10 | 2 | 0.04 | + | 45.83 | 1.20E-03 | 3.60E-02 |
| catecholamine catabolic process (GO:0042424) | 10 | 2 | 0.04 | + | 45.83 | 1.20E-03 | 3.61E-02 |
| phospholipid dephosphorylation (GO:0046839) | 45 | 3 | 0.2 | + | 15.28 | 1.19E-03 | 3.61E-02 |
| positive regulation of mitophagy (GO:1901526) | 10 | 2 | 0.04 | + | 45.83 | 1.20E-03 | 3.61E-02 |
| positive regulation of cellular respiration (GO:1901857) | 10 | 2 | 0.04 | + | 45.83 | 1.20E-03 | 3.62E-02 |
| negative regulation of macromolecule metabolic process (GO:0010605) | 2867 | 24 | 12.51 | + | 1.92 | 1.24E-03 | 3.73E-02 |
| regulation of cyclin-dependent protein kinase activity (GO:1904029) | 104 | 4 | 0.45 | + | 8.81 | 1.27E-03 | 3.79E-02 |
| cellular component biogenesis (GO:0044085) | 2652 | 23 | 11.57 | + | 1.99 | 1.28E-03 | 3.80E-02 |
| regulation of system process (GO:0044057) | 597 | 9 | 2.61 | + | 3.45 | 1.28E-03 | 3.81E-02 |
| positive regulation of apoptotic signaling pathway (GO:2001235) | 180 | 5 | 0.79 | + | 6.36 | 1.28E-03 | 3.81E-02 |
| positive regulation of signaling (GO:0023056) | 1867 | 18 | 8.15 | + | 2.21 | 1.30E-03 | 3.86E-02 |
| signal release from synapse (GO:0099643) | 105 | 4 | 0.46 | + | 8.73 | 1.31E-03 | 3.87E-02 |
| neurotransmitter secretion (GO:0007269) | 105 | 4 | 0.46 | + | 8.73 | 1.31E-03 | 3.88E-02 |
| regulation of developmental process (GO:0050793) | 2668 | 23 | 11.64 | + | 1.98 | 1.33E-03 | 3.91E-02 |
| regulation of dendritic spine morphogenesis (GO:0061001) | 47 | 3 | 0.21 | + | 14.63 | 1.34E-03 | 3.94E-02 |
| mitochondrion localization (GO:0051646) | 47 | 3 | 0.21 | + | 14.63 | 1.34E-03 | 3.95E-02 |
| response to endogenous stimulus (GO:0009719) | 1433 | 15 | 6.25 | + | 2.4 | 1.38E-03 | 4.05E-02 |
| positive regulation of ATP metabolic process (GO:1903580) | 48 | 3 | 0.21 | + | 14.32 | 1.42E-03 | 4.07E-02 |
| positive regulation of oxidative phosphorylation (GO:1903862) | 11 | 2 | 0.05 | + | 41.66 | 1.41E-03 | 4.07E-02 |
| response to amyloid-beta (GO:1904645) | 48 | 3 | 0.21 | + | 14.32 | 1.42E-03 | 4.08E-02 |
| sterol catabolic process (GO:0016127) | 11 | 2 | 0.05 | + | 41.66 | 1.41E-03 | 4.08E-02 |
| regulation of vacuole organization (GO:0044088) | 48 | 3 | 0.21 | + | 14.32 | 1.42E-03 | 4.09E-02 |
| regulation of neuromuscular junction development (GO:1904396) | 11 | 2 | 0.05 | + | 41.66 | 1.41E-03 | 4.09E-02 |
| cellular process (GO:0009987) | 15626 | 81 | 68.2 | + | 1.19 | 1.42E-03 | 4.09E-02 |
| skin morphogenesis (GO:0043589) | 11 | 2 | 0.05 | + | 41.66 | 1.41E-03 | 4.10E-02 |
| negative regulation of G1/S transition of mitotic cell cycle (GO:2000134) | 107 | 4 | 0.47 | + | 8.57 | 1.40E-03 | 4.10E-02 |
| regulation of supramolecular fiber organization (GO:1902903) | 376 | 7 | 1.64 | + | 4.27 | 1.41E-03 | 4.10E-02 |
| regulation of dopamine receptor signaling pathway (GO:0060159) | 11 | 2 | 0.05 | + | 41.66 | 1.41E-03 | 4.10E-02 |
| regulation of synapse assembly (GO:0051963) | 107 | 4 | 0.47 | + | 8.57 | 1.40E-03 | 4.10E-02 |
| cholesterol catabolic process (GO:0006707) | 11 | 2 | 0.05 | + | 41.66 | 1.41E-03 | 4.11E-02 |
| organic cyclic compound catabolic process (GO:1901361) | 489 | 8 | 2.13 | + | 3.75 | 1.44E-03 | 4.13E-02 |
| glycolipid metabolic process (GO:0006664) | 108 | 4 | 0.47 | + | 8.49 | 1.45E-03 | 4.14E-02 |
| cellular biosynthetic process (GO:0044249) | 2701 | 23 | 11.79 | + | 1.95 | 1.45E-03 | 4.14E-02 |
| regulation of hormone secretion (GO:0046883) | 277 | 6 | 1.21 | + | 4.96 | 1.47E-03 | 4.19E-02 |
| liposaccharide metabolic process (GO:1903509) | 109 | 4 | 0.48 | + | 8.41 | 1.50E-03 | 4.24E-02 |
| response to alkaloid (GO:0043279) | 109 | 4 | 0.48 | + | 8.41 | 1.50E-03 | 4.25E-02 |
| regulation of release of cytochrome c from mitochondria (GO:0090199) | 49 | 3 | 0.21 | + | 14.03 | 1.50E-03 | 4.25E-02 |
| cellular component assembly (GO:0022607) | 2391 | 21 | 10.44 | + | 2.01 | 1.51E-03 | 4.27E-02 |
| negative regulation of cell cycle G1/S phase transition (GO:1902807) | 110 | 4 | 0.48 | + | 8.33 | 1.55E-03 | 4.34E-02 |
| positive regulation of proteasomal protein catabolic process (GO:1901800) | 110 | 4 | 0.48 | + | 8.33 | 1.55E-03 | 4.35E-02 |
| regulation of transmembrane transporter activity (GO:0022898) | 280 | 6 | 1.22 | + | 4.91 | 1.55E-03 | 4.36E-02 |
| phosphatidylinositol biosynthetic process (GO:0006661) | 110 | 4 | 0.48 | + | 8.33 | 1.55E-03 | 4.36E-02 |
| regulation of hydrolase activity (GO:0051336) | 1302 | 14 | 5.68 | + | 2.46 | 1.58E-03 | 4.41E-02 |
| protein stabilization (GO:0050821) | 189 | 5 | 0.82 | + | 6.06 | 1.58E-03 | 4.42E-02 |
| negative regulation of secretion (GO:0051048) | 190 | 5 | 0.83 | + | 6.03 | 1.62E-03 | 4.51E-02 |
| regulation of dendritic spine maintenance (GO:1902950) | 12 | 2 | 0.05 | + | 38.19 | 1.64E-03 | 4.53E-02 |
| postsynaptic membrane assembly (GO:0097104) | 12 | 2 | 0.05 | + | 38.19 | 1.64E-03 | 4.53E-02 |
| cellular transition metal ion homeostasis (GO:0046916) | 112 | 4 | 0.49 | + | 8.18 | 1.65E-03 | 4.53E-02 |
| parental behavior (GO:0060746) | 12 | 2 | 0.05 | + | 38.19 | 1.64E-03 | 4.54E-02 |
| maternal behavior (GO:0042711) | 12 | 2 | 0.05 | + | 38.19 | 1.64E-03 | 4.55E-02 |
| amyloid fibril formation (GO:1990000) | 12 | 2 | 0.05 | + | 38.19 | 1.64E-03 | 4.56E-02 |
| intestinal lipid absorption (GO:0098856) | 12 | 2 | 0.05 | + | 38.19 | 1.64E-03 | 4.57E-02 |
| negative regulation of excitatory postsynaptic potential (GO:0090394) | 12 | 2 | 0.05 | + | 38.19 | 1.64E-03 | 4.57E-02 |
| cell junction assembly (GO:0034329) | 284 | 6 | 1.24 | + | 4.84 | 1.67E-03 | 4.58E-02 |
| social behavior (GO:0035176) | 51 | 3 | 0.22 | + | 13.48 | 1.68E-03 | 4.58E-02 |
| protein destabilization (GO:0031648) | 51 | 3 | 0.22 | + | 13.48 | 1.68E-03 | 4.59E-02 |
| intraspecies interaction between organisms (GO:0051703) | 51 | 3 | 0.22 | + | 13.48 | 1.68E-03 | 4.60E-02 |
| central nervous system neuron differentiation (GO:0021953) | 192 | 5 | 0.84 | + | 5.97 | 1.69E-03 | 4.62E-02 |
| regulation of synaptic plasticity (GO:0048167) | 193 | 5 | 0.84 | + | 5.94 | 1.73E-03 | 4.71E-02 |
| positive regulation of cytosolic calcium ion concentration (GO:0007204) | 287 | 6 | 1.25 | + | 4.79 | 1.76E-03 | 4.77E-02 |
| modulation of process of other organism involved in symbiotic interaction (GO:0051817) | 114 | 4 | 0.5 | + | 8.04 | 1.76E-03 | 4.77E-02 |
| negative regulation of response to endoplasmic reticulum stress (GO:1903573) | 52 | 3 | 0.23 | + | 13.22 | 1.77E-03 | 4.79E-02 |
| phospholipid metabolic process (GO:0006644) | 392 | 7 | 1.71 | + | 4.09 | 1.77E-03 | 4.80E-02 |
| regulation of body fluid levels (GO:0050878) | 506 | 8 | 2.21 | + | 3.62 | 1.78E-03 | 4.81E-02 |
| glial cell development (GO:0021782) | 115 | 4 | 0.5 | + | 7.97 | 1.81E-03 | 4.88E-02 |
| positive regulation of peptidase activity (GO:0010952) | 196 | 5 | 0.86 | + | 5.85 | 1.85E-03 | 4.97E-02 |
| macrophage activation (GO:0042116) | 53 | 3 | 0.23 | + | 12.97 | 1.86E-03 | 4.99E-02 |
| regulation of microtubule polymerization (GO:0031113) | 53 | 3 | 0.23 | + | 12.97 | 1.86E-03 | 5.00E-02 |


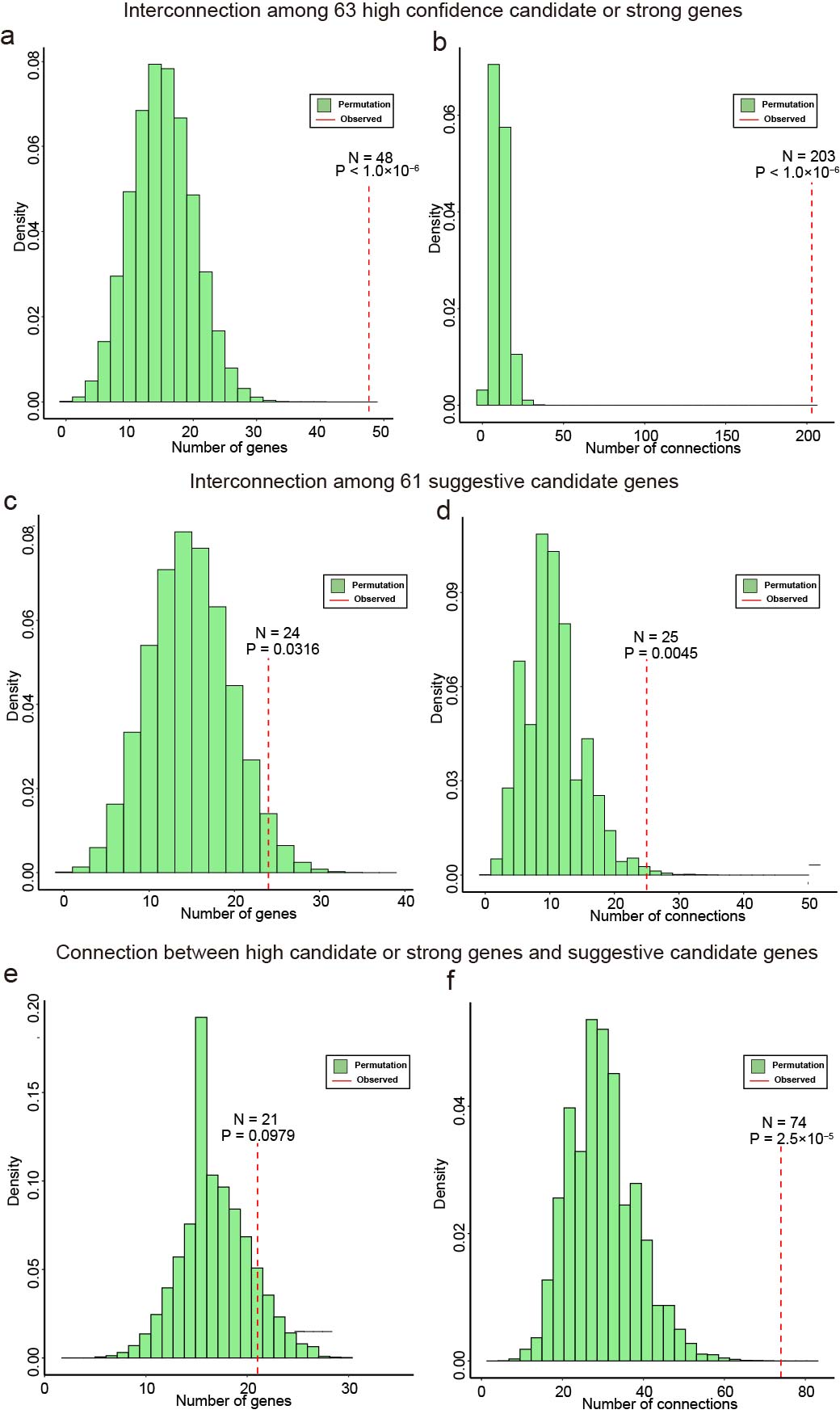


**Supplementary Figure 1. The interconnectivities among PD-associated genes accessed by permutation tests.** (a-b) The interconnectivities among high confidence/strong PD-associated genes. (c-d) The interconnectivities among suggestive associated genes. (e-f) The connection between high confidence/strong PD-associated genes and suggestive associated genes.


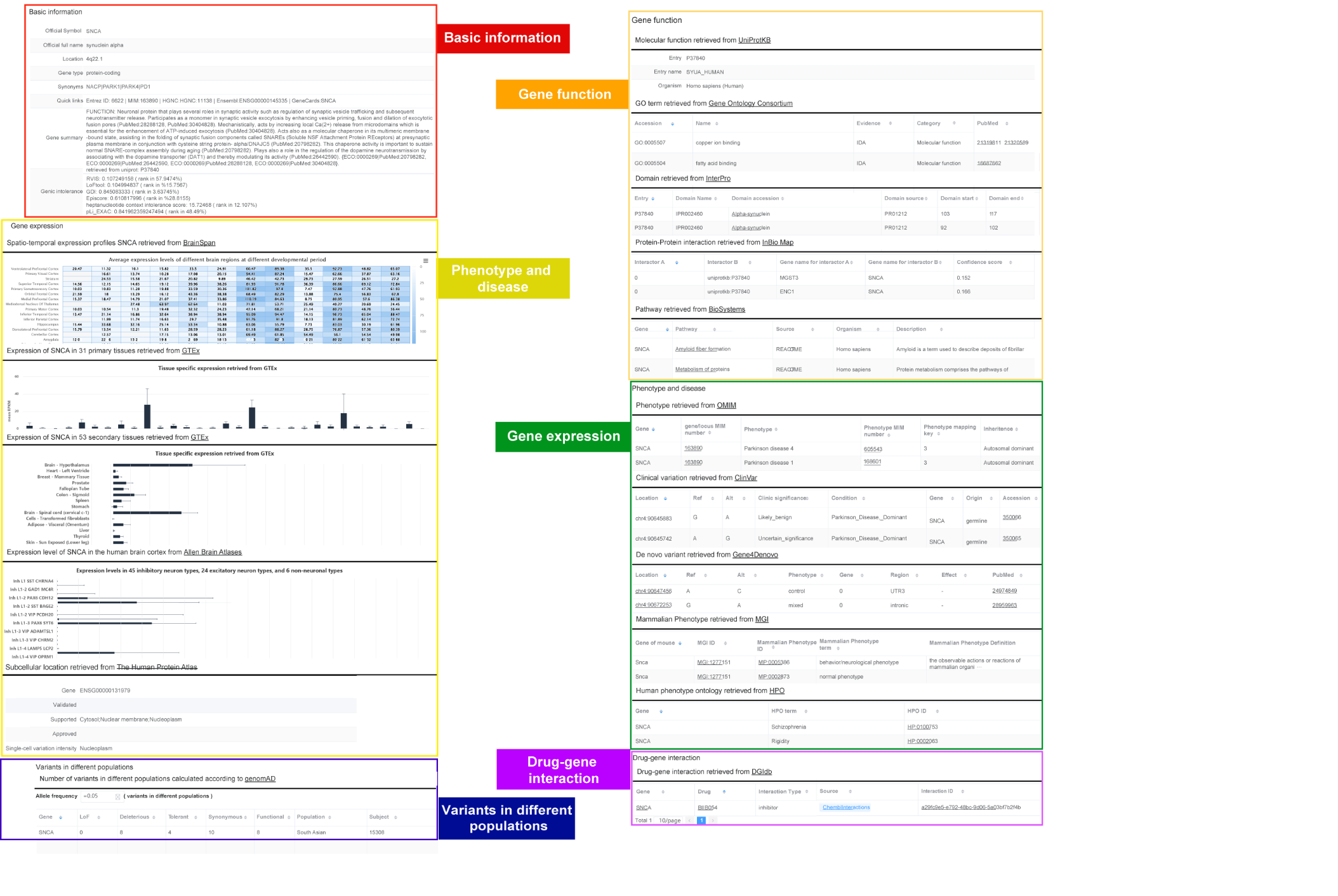


**Supplementary Figure 2. Snapshot of gene-level implications in Gene4PD.** Six sections, including ‘basic information’, ‘gene functions’, ‘phenotype and disease’, ‘gene expression’, ‘Variants in different population’, ‘Drug-gene interaction’ are illustrated in gene-level implications.


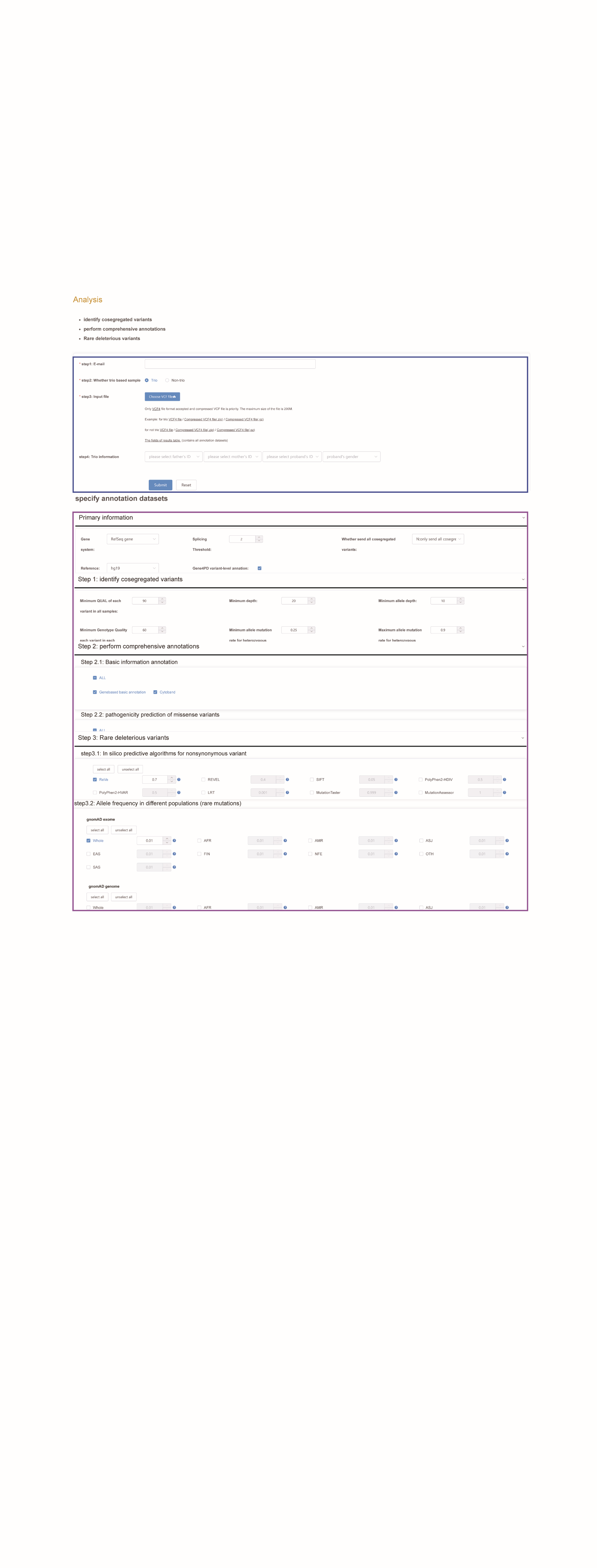


**Supplementary Figure 3. Snapshot of analysis panel in Gene4PD.** Two main sections, including inputting cosegregated variants panel, and choosing specify annotation datasets were illustrated in the analysis panel.
